# Ribosome Proteins Represented by RPL27A Mark the Development and Metastasis of Triple-Negative Breast Cancer in Mouse and Human

**DOI:** 10.3389/fcell.2021.716730

**Published:** 2021-08-23

**Authors:** Weipeng Zhao, Xichuan Li, Weiqi Nian, Jun Wang, Xiaorui Wang, Linlin Sun, Ye Zhu, Zhongsheng Tong

**Affiliations:** ^1^Key Laboratory of Cancer Prevention and Therapy, Department of Breast Cancer, Tianjin Medical University Cancer Institute and Hospital, National Clinical Research Center for Cancer, Tianjin, China; ^2^Tianjin Key Laboratory of Animal and Plant Resistance, College of Life Sciences, Tianjin Normal University, Tianjin, China; ^3^Chongqing Key Laboratory of Translational Research for Cancer Metastasis and Individualized Treatment, Chongqing University Cancer Hospital, Chongqing, China; ^4^Department of Oncology, The First Affiliated Hospital of Shandong First Medical University, Jinan, China

**Keywords:** single cell RNA-sequencing, triple negative breast cancer, ribosomal protein, RPL27A, EIF2 signaling

## Abstract

Triple-negative breast cancer (TNBC) is known to have a poor prognosis and limited treatment options. The lack of targeted therapies and poor prognosis of patients with TNBC have made it urgent to discover novel critical diagnosis and therapeutic targets in the TNBC field. Here, in the current study, we integrated the single-cell RNA-sequencing (scRNA-seq) data from four normal mouse mammary tissues and four mouse breast tumors. Comparative analysis was conducted to identify the gene profiles of normal epithelial cells and cancer cells at different models. Surprisingly, two ribosomal protein genes, Rpl27a and Rpl15, were significantly upregulated in the cancer cells in all the TNBC models. Next, we accessed the scRNA-seq data from human primary and metastatic TNBC tissues, and comparative analysis revealed gene profiles of human primary and metastatic TNBC cancer cells. Ribosomal protein genes, represented by RPL27A and RPL15, showed significantly upregulated expression in metastatic TNBC cancer cells. Pathway analysis on the upregulated genes of the metastatic TNBC cancer cells identified the key regulators and signaling pathways that were driving the metastasis of the TNBC cancer cells. Specifically, EIF2 signaling was significantly activated, and major member genes of this signaling pathway were upregulated. *In vitro* study revealed that targeting RPL27A or EIF2 signaling in a TNBC cell line, MDA-MB-231, significantly reduced cell migration and invasion. Altogether, these data suggested that the RPL27A gene is conducting critical functions in TNBC cancer development and metastasis and is a potential therapeutic target for TNBC.

## Introduction

Triple-negative breast cancer (TNBC) is defined as a type of breast cancer deficient of expression of estrogen receptor (ER), progesterone receptor (PR), and HER2 protein and present a particularly challenging therapeutic target due to their highly invasive nature and relatively low response to therapeutics (Garrido-Castro et al., 2019). In comparison to other types of breast cancer, TNBC is characterized by its aggressive behavior, high metastatic potential, poor prognosis, and proneness to relapse (Yin et al., 2020). The definition of TNBC applies to breast cancer that lack the expression of ER, PR, and HER2, and all of these are molecular targets of therapeutic agents. Nevertheless, chemotherapy is still the primary established treatment option for patients with early-stage TNBC and those with advanced-stage TNBC (Bianchini et al., 2016), but the efficacy of conventional postoperative adjuvant chemoradiotherapy is poor. The residual metastatic lesions frequently lead to tumor recurrence after surgery (Yin et al., 2020). Due to the special molecular and genetic phenotypes, TNBC is not sensitive to endocrine therapy or molecular targeted therapy. Bevacizumab has been added to chemotherapeutic drugs to treat TNBC in some countries, although there is not any demonstrated overall survival benefit (Collignon et al., 2016). Many other standard adjuvant and neoadjuvant regimens including anthracyclines, cyclophosphamide, and taxanes are used, but their efficiencies are yet to be determined (Collignon et al., 2016). Although increasing research is going on to identify specific targets and develop additional and better systemic treatment options, there are no approved targeted therapies for TNBC up to now.

Ribosomal proteins are many of the proteins that, in conjunction with rRNA, make up the ribosomal subunits involved in the cellular process of translation (Zhou et al., 2015). Beyond their essential roles in ribosome assembly and protein translation, ribosome-independent functions of ribosomal proteins have also been greatly appreciated, especially in the study achievements connecting cancer diagnosis and therapy (Penzo et al., 2019).

A number of tumor suppressors and oncogenic proteins often control the progression of cancer cells by regulating ribosome biogenesis and global protein synthesis (Ruggero and Pandolfi, 2003; Silvera et al., 2010). Interestingly, individual ribosome-independent ribosomal proteins also conduct critical functions in tumorigenesis, especially in breast cancer. For instance, upregulation of RPL19 induces ER stress, resulting in increased sensitivity to ER stress and enhanced cell death in MCF7 breast cancer cells (Hong et al., 2014). RPS16 and TNFSF10 as two direct targets of miR-7641 and many other ribosomal proteins that are frequently co-expressed with RPS16 in breast cancer are also deregulated by miR-7641. This is making miR-7641a potential targeting factor to improve the efficacy of cancer therapy (Reza et al., 2017). Targeting RPL39 and MLF2 reduces tumor initiation and metastasis in breast cancer by inhibiting nitric oxide synthase signaling. RPL5 downregulation is also associated with breast cancer cell proliferation and tumor progression in transgenic mice and human tumor xenograft mouse models (Fancello et al., 2017). All these *in vitro* studies shed light on the functions of ribosomal proteins on breast cancer phenotypes, but few were TNBC specific. A more interesting study identified two previously unidentified cancer genes, RPL39 and MLF2, by selective shRNA knockdown of genes from this tumorigenic signature, which impacted breast cancer stem cell self-renewal and lung metastases. Knockdown of these genes in TNBC models significantly reduced primary tumor growth, as well as metastasis (Dave et al., 2014). Another most recent study revealed that RPL15-overexpressing circulating tumor cells markedly increased metastatic burden in mice.

Interestingly, in the current study, through a complete research on the published single-cell RNA-sequencing (scRNA-seq) databases, by comparative analysis of normal mouse breast epithelial cell and TNBC cancer cells in mouse models, as well human primary and brain metastatic TNBC at single cell levels, we revealed that RPL15 was significantly increased in mouse tumor cells and metastatic TNBC compared to corresponding controls. A more interesting gene is RPL27A, which also showed ectopic expression and potentially promoted TNBC development and metastasis *via* the EIF2 signaling pathway. These findings revealed a novel molecular biomarker and a potential related signaling pathway for the prognosis and response prediction of TNBC to traditional therapy and new molecular target agents.

## Materials and Methods

### Reuse of scRNA-Seq Dataset

Single-cell RNA-sequencing on mammary epithelial cells across four developmental stages (nulliparous, mid-gestation, lactation, and post involution) were described by the original report (Bach et al., 2017). We accessed the processed data of this scRNA-seq dataset from the Gene Expression Omnibus under accession number GSE106273 and raw data of the scRNA-seq from Sequence Read Archive (SRA) under accession number PRJNA416110 for our analysis. scRNA-seq data on different mouse models of breast cancer were retrieved from GEO under accession number GSE123366 and SRA under accession number PRJNA508501 (Yeo et al., 2020). scRNA-seq data on primary TNBC were retrieved from GEO under accession number GSE118389 and SRA under accession number PRJNA485429 (Karaayvaz et al., 2018), and scRNA-seq data on brain metastatic TNBC were retrieved from GEO under accession number GSE143423 and SRA under accession number PRJNA600483.

### scRNA-Seq Analysis and Differentially Expressed Gene Identification

RStudio software package Seurat was used for the analysis of matrix data of scRNA-seq. Sample objects were integrated or merged by either sample individuals or their disease status. Anchors were generated, listing all the Seurat objects as input, and integrated/merged data were created for integration. In the integrated/merged Seurat object, “nFeature_RNA,” “nCount_RNA,” and “percent.MT” were defined to represent number of genes, number of transcripts, and percentage of mitochondrial initiated genes in each cell of each sample, respectively. Quality control (QC) was performed by removing the low-quality events and doublets by excluding the cells with extreme nCount_RNA or nFeature_RNA and high percent.MT.

Canonical cell-type markers were used to identify and define the major cell types. For the epithelial/cancer cell lineage, we used common epithelial cell marker genes [CDH1 (Cdh1), KRT18 (Krt18), and KRT5 (Krt5)] and endothelial cell marker genes [CDH5 (Cdh5), PECAM1 (Pecam1), and EMCN (Emcn)]. Total immune cells were identified by PTPRC (Ptprc), CD68 (Cd68), and CSF1R (Csf1r). For the mesenchymal cell component, COL1A1 (Col1a1), COL1A2 (Col1a2), COL3A1 (Col3a1), and FAP were used. The cancer/epithelial cell clusters were extracted from other cell types and re-clustered for further analysis.

### Pseudotime Analysis With Slingshot and Ingenuity Pathway Analysis

The scRNA-seq data of epithelial/cancer cells were loaded into Slingshot packages, and major clusters were identified for pseudotime analysis. Analysis on inferred trajectory and principal curves demonstrated smoothed representations of each subcluster. Output principal curves were smoothed representations of each disease status, and pseudotime values were computed by projecting the cells onto the principal curves. For the Ingenuity Pathway Analysis (IPA), differentially expressed genes of each disease status were identified and were loaded into the IPA client (QIAGEN) to demonstrate the key ingenuity canonical pathways and upstream regulators. The ingenuity canonical pathways, upstream regulators, and graphical summary of the regulatory networks were exported from the IPA.

### Cell Culture and Knockdown Assay

MDA-MB-231 cell (RRID: CVCL_0062) was purchased from American Type Culture Collection (ATCC) and was cultured in L-15 medium supplemented with 10% FBS, penicillin (100 U/ml), and streptomycin (100 μg/ml). The cells have been authenticated using STR profiling within the last 3 years. All experiments were performed with mycoplasma-free cells. MDA-MB-231 cells were cultured in six-well plates to 30–60% confluency and shRPL27A (Santa Cruz Biotechnology), or scramble lentiviral particles were mixed in complete medium together with polybrene (10 μg/ml). Cells were cultured with lentiviral particles for 4 h, and then the medium was changed. Cells were cultured until confluency and were passaged for further use. For the EIF2 inhibitor assay, 20 and 50 μm Sal003 were used, and the same amount of DMSO was used as controls.

### RNA Extraction and Quantitative Real-Time PCR

The RNeasy Mini kit (QIAGEN) was used to extract total RNA from samples following transfection according to the manufacturer’s protocols. The expression of human RPL27A was determined using the SYBR Green Master Mix kit (Roche). Human GAPDH was used as an internal control gene, and relative expression levels were calculated by using the 2^–ΔΔCt^ method. The sequences of specific primer pairs are described below: RPL27A-F 5′-ACGGGTGAATGCTGCTAAAA-3′ and RPL27A-R 5′-GAAGAATTTGGCCTTCACGA-3′; RPL37A-F 5′-ACATGGCCAAACGTACCA-3′ and RPL37A-R 5′-TGCTG GCTGATTTCAATTTT-3′; RPL30-F 5′-ATGGCCAAACGTA CCAAG-3′ and RPL30-R 5′-AAGTGTACTTGGCGTGCTG-3′; RPL39 primer (Bio-Rad, qHsaCED0038310); RPL36 primer (Bio-Rad, qHsaCED0038433); RPL36A primer (Bio-Rad, qHsaCED0044302); RPL15 primer (Bio-Rad, qHsaCED0038184); RPL21 primer (Bio-Rad, qHsaCED0038684); RPL38 primer (Bio-Rad, qHsaCID0038174); RPS7 primer (Bio-Rad, qHsaCID0038168); RPS15A primer (Bio-Rad, qHsaCED0038046); RPS17 primer (Bio-Rad, qHsaCED0041771); RPS21 primer (Bio-Rad, qHsaCID0023899); RPS27A primer (Bio-Rad, qHsaCED0038707); RPS29 primer (Bio-Rad, qHsaCED0038808); and GAPDH-F 5′-AGCCACATCGCTCAGACAC-3′ and GAPDH-R 5′-GCCCA ATACGACCAAATCC-3′.

### Western Blotting

RIPA buffer (Thermo Fisher Scientific) and Protease and Phosphatase Inhibitor (Thermo Fisher Scientific) were used to extract proteins. The primary antibodies against antibody for RPL27A (NBP2-38025, Novus Biologicals) and GAPDH (NB300-221, Novus Biologicals) and secondary antibodies were anti-mouse IgG-HRP (7076S, Cell Signaling Technology) and anti-rabbit IgG-HRP (7074S, Cell Signaling Technology). The protein blots were visualized using an ECL Western Blotting Substrate (Thermo Fisher Scientific).

### Migration and Invasion Assay

Twenty-four-well chambers (Corning) were used or migration assay, and 24-well Matrigel invasion chambers (BD Biosciences) were used for invasion assay. MDA-MB-231 cells (5 × 10^4^) were placed in 100-μl serum-free medium in the migration/migration chamber, and 750 μl complete medium was placed into the lower wells. After culturing for 24 h, cells were fixed with 100% methanol for 20 min and stained with Trypan blue for 30 min. Non-migrating cells on the upper side of the filter were removed with cotton swabs. Migration and invasion were quantified by counting the number of cells on the lower surface of the filter.

### Statistical Analysis

The cell number of each cluster or each disease status was determined in Seurat. Student’s two-tailed *t*-test was used for comparing differences between two groups, and *p* < 0.05 was considered statistically significant: ^∗^*p* < 0.05; ^∗∗^*p* < 0.01; ^∗∗∗^*p* < 0.001, ^****^p < 0.0001. The cell numbers and cell percentages were calculated and were plotted using GraphPad Prism 8.

### Data and Code Availability

All the scRNA-seq data are available in GEO (accession numbers are above), and the codes regarding to the data analysis are accessible upon request.

## Results

### Single-Cell Profiling of TNBC Cells and Normal Breast Epithelial Cells

To gain insights into the heterogeneity within mammary tumors, a recent study performed single-cell transcriptional profiling of breast tumor cells from four mouse models (Yeo et al., 2020). The MMTV-Neu (NEU) tumor was ER^–^/PR^–^/HER2^+^, while the MMTV-PyMT (FF99WT) tumor was ER^–^/PR^–^/HER2^low^; the BRCA1-null (BRCA1) tumor which mimics basal-like breast cancers was ER^–^/PR^–^/HER^–^, and the 4T1 transplant tumor was a known ER^–^/PR^–^/HER^–^ mouse model. To study the gene profiles of the tumor cells in these four models, we reaccessed the scRNA-seq data from GEO. We first integrated all the scRNA-seq data of different samples and models and corrected the batch effects. Cell qualities in each sample were visualized by violin plots. Cells that met the requirement were retained, and cells with extreme nFeature_RNA and nCount_RNA or high percent.MT were removed (Supplementary Figures 1A,B). After clustering, the distribution of each sample (Supplementary Figure 1C) and each model (Supplementary Figure 1D) was visualized by Uniform Manifold Approximation and Projection (UMAP). In total, 14 major cell clusters were identified, and the clusters of each model were also visualized (Supplementary Figure 1E).

We then checked the expression of the canonical cell-type markers: cancer/epithelial cell marker genes (Cdh1, Krt18, and Krt5); mesenchymal cell marker genes (Col1a1, Col1a2, and Col3a1); immune cell marker genes (Ptprc, Cd68, and Csf1r); and endothelial cell marker genes (Pecam1, Emcn, and Cdh5) (Supplementary Figure 1F). Based on the transcripts of these genes, we accordingly identified the cancer cells, mesenchymal/endothelial cells in the integrated data, and each tumor model (Supplementary Figures 1G,H). Then the cancer cells were subset from the integrated data and re-clustered. Cells from each sample (Supplementary Figure 2A) and model (Supplementary Figure 2B) were visualized to confirm the batch correction. Although some cells showed biased distribution in a few clusters of the integrated data, suggesting different cell numbers in some samples in these clusters, most of the cells were evenly distributed (Supplementary Figures 2C,D).

We next determined the transcript of basal marker Krt5 and the luminal marker Krt18. Notably, all the cancer cells from the four models showed high Krt18 expression, and only some cells from the BRCA1 model, a basal-like breast cancer model, showed a detectable Krt5 transcript (Supplementary Figure 2E). We also checked the transcriptional levels of ER genes, PR genes, and HER2 (Erbb2) and found rare transcripts in the cancer cells of all the tumor models, even in the NEU model (Supplementary Figure 2F), suggesting that these were good models to mimic TNBC.

As the control of the tumor cells, we then accessed another scRNA-seq dataset on normal mouse mammary epithelial cells at four developmental stages: nulliparous, mid-gestation, lactation, and post involution (Bach et al., 2017). Similarly, sample integration and QC were performed on these data (Supplementary Figures 3A,B). After clustering, cells from different samples and developmental stages, as well as the cell clusters, were visualized by UMAP (Supplementary Figures 3C–E). The major cell types, including epithelial cells, mesenchymal cells, immune cells, and endothelial cells, were identified by checking canonical cell-type marker genes (Supplementary Figures 3F–H). The epithelial cells were then subset and re-clustered, and epithelial cells from different stages (Supplementary Figures 4A,B) showed biased distribution in the clusters (Supplementary Figures 4C,D), suggesting that the gene profiles in different stages differed significantly, confirming the findings in the original study (Bach et al., 2017). The basal marker Krt5 and the luminal marker Krt18 were used to confirm this (Supplementary Figures 4E,F). It was clear that the epithelial cells in nulliparous and mid-gestation stages were composed of both Krt18^+^ and Krt5^+^ cells, and in the lactation stage, the epithelial cells were mostly Krt5^+^ cells, while in the post involution stage, the epithelial cells were mainly Krt18^+^ cells (Supplementary Figures 4E,F).

### Breast Cancer Cell Profiling Compared to Normal Mammary Epithelial Cells

**FIGURE 1:**
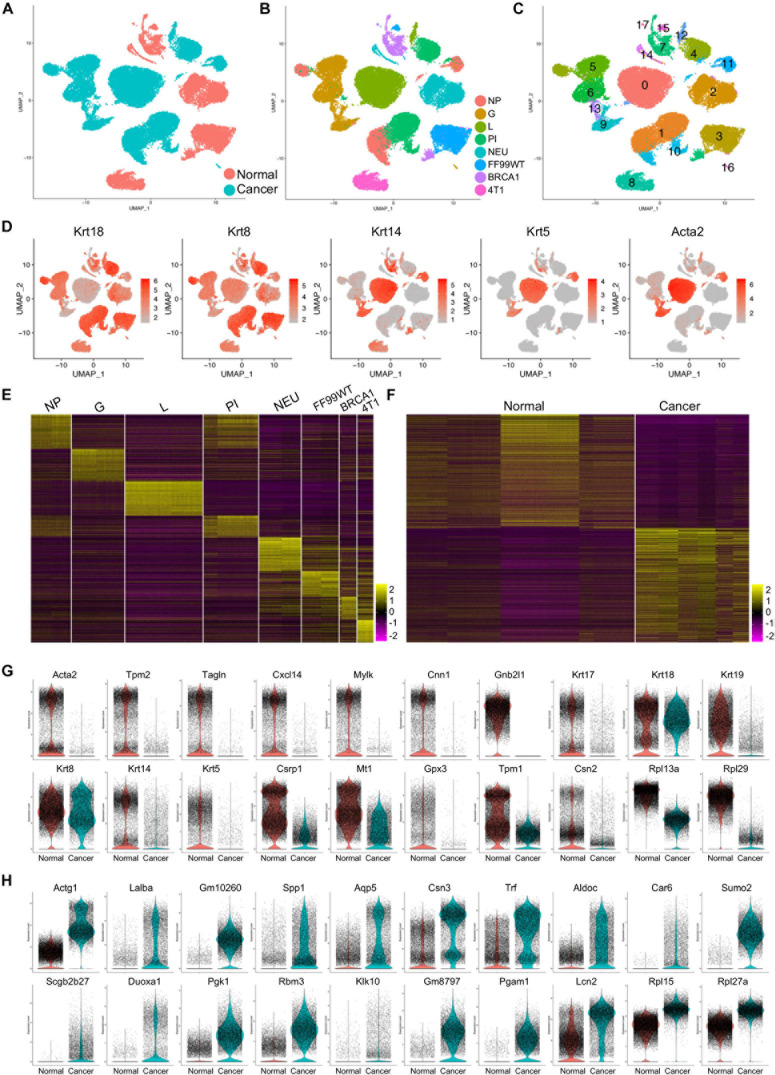
Gene expression patterns of the cancer cells and normal epithelial cells. **(A,B)** Distribution of normal and cancer cells **(A)** and cells from different developmental stages and mouse models **(B)** in the merged data. **(C)** Identification of the major cell clusters in the merged scRNA-seq data. **(D)** Transcription of the canonical luminal marker genes and basal marker genes in the merged data. **(E,F)** Heatmap visualization of the top 100 genes of cells from each developmental stage and breast cancer mouse model **(E)** and the top 500 genes of the normal epithelial cells and cancer cells **(F)**. **(G,H)** Violin plot presentation of the top 20 genes upregulated in normal **(G)** or cancer **(H)** cells.

The cancer cells were subset from the tumor models, and the normal epithelial cells were subset from normal mammary datasets, and then these cells were merged (Figure 1A). Due to the gene profile differences, the cancer cells and normal epithelial cells showed distinct clusters (Figure 1B). The integrated data were clustered, and 17 major cell clusters were identified (Figure 1C). Most of the clusters showed specific expression of either the luminal markers Krt18 and Krt8 or basal markers Krt14, Krt5, and Acta2 (Figure 1D).

To determine the gene profiles of the cells of different developmental stages/models, we performed comparative analysis on the scRNA-seq data, and the top 100 genes were presented by heatmap, suggesting distinct gene profiling patterns in each developmental stage/model (Figure 1E). Further comparative analysis was used to identify the profiling difference of normal epithelial cells and cancer cells. The top 500 genes in the total normal epithelial cells and total cancer cells were visualized by heatmap (Figure 1F). The top 20 upregulated genes in normal epithelial cells (Figure 1G) and in cancer cells (Figure 1H) were visualized by violin plots. Most of the top genes identified in normal epithelial cells, including Acta2 (Weymouth et al., 2012), Mylk, Cnn1 (Bresson et al., 2018), and multiple Krt genes (Kendrick et al., 2008), were associated with normal development and functions of mammary gland, such as maintenance of lactation, mammary stem cell function, and lineage commitment determination. Notably, two function-unidentified ribosome protein genes, Rpl13a and Rpl29, were significantly downregulated in cancer cells (Figure 1G and Supplementary Figure 5A). Of the top genes upregulated in the cancer cells, most were reported to be breast cancer-promoting genes. Specifically, AGTG1 is one of the recurrent mutated genes in breast cancer (Ciriello et al., 2015). Lalba is reported to not be expressed in healthy tissues except during lactation, while it is expressed at high levels in a subset of human breast cancers, especially TNBC (Tuohy, 2014). Higher SPP1 gene expression in primary tumors was found to be associated with risk of recurrence in ER^+^ breast cancer among patients with endocrine treatment (Gothlin Eremo et al., 2020). Modification of Sumo2 was associated with the progression and metastasis of breast cancer (Subramonian et al., 2014). Other genes were associated with cell adhesion and migration of breast cancer cell [DUOXA1 (Ostrakhovitch and Li, 2010) and PGAM1 (Zhang et al., 2017)], prognosis of breast cancer [PGK1 (Fu et al., 2018) and KLK10 (Kioulafa et al., 2009)], or clinical outcome in breast cancer [RBM3 (Jogi et al., 2009) and AQP5 (Lee et al., 2014)]. Another gene was Rpl15, which was recently reported to markedly increase metastatic burden in TNBC mouse models (Ebright et al., 2020). This was further confirmed in our study by the data that an increased expression of Rpl15 gene was found in the cancer cells. A more interesting gene was Rpl27a, which also showed significantly increased transcript in cancer cell (Figure 1H and Supplementary Figure 5A) and whose functions had rarely been studied in diseases or cancers.

### Rpl27a Is Upregulated in Cancer Cells of Murine TNBC Models

**FIGURE 2:**
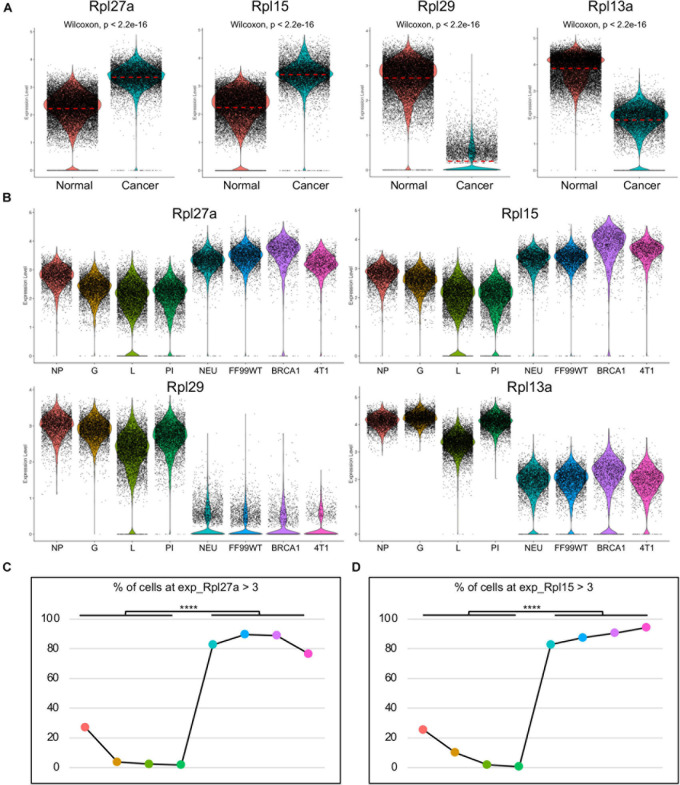
Upregulated Rpl27a and Rpl15 in cancer cells of the mouse models. **(A)** Violin plots showing transcription levels of Rpl27a, Rpl15, Rpl29, and Rpl13a in normal epithelial cells and breast cancer cells. **(B)** Visualization of transcription levels of Rpl27a, Rpl15, Rpl29, and Rpl13a in epithelial cells of each developmental stage and breast cancer cells of each mouse model by split violin plots. **(C,D)** Quantification of the percentage of cells with Rpl27a > 3 **(C)** or Rpl15 > 3 **(D)** in each developmental stage and breast cancer mouse model. ****p < 0.0001.

To better clarify the expression level of Rpl27a, the transcripts were visualized by both violin plots and UMAPs (Figure 2A and Supplementary Figure 5A). The transcripts of Rpl27a in split violin plots (Figure 2B) and UMAPs (Supplementary Figure 6) further confirmed the significantly upregulated transcription in cancer cells of all the cancer models compared to normal epithelial cells in all the mammary developmental stages. Consistently, Rpl15 was also significantly upregulated and Rpl29 and Rpl13a were downregulated (Figures 2A,B and Supplementary Figures 5A, 6). To further quantify the expression of Rpl27a, we calculated the percentage of the cells with Rpl27a expression at Rpl27a > 3. Notably, percentages of cells with Rpl27a expression among the four tumor models were comparable but were significantly higher than those of normal mammary epithelial cells of developmental stages (Figure 2C). This was also true for Rpl15 (Figure 2D). On the contrary, percentages of Rpl29-expressing and Rpl13a-expressing cells were notably decreased in cancer models (Supplementary Figures 5B,C). These data confirmed that Rpl27a was potentially a diagnosis biomarker for TNBC in the mouse models, but its expression levels in human breast cancer and its functions and underlying mechanisms needed to be further determined.

### Gene Expression Patterns of Primary and Metastatic TNBC Cells

To validate the expression of Rpl27a in human TNBC tumor cells, we first accessed a published scRNA-seq data on primary human TNBC tumors (Karaayvaz et al., 2018). Similar to the analysis of mouse scRNA-seq data, we performed QC to remove the low-quality cells (Supplementary Figures 7A,B). High-quality cells were then clustered (Supplementary Figure 7C), and major cell types were identified by canonical cell-type marker expression (Supplementary Figures 7D,E). The cancer cells were subset for further analysis (Supplementary Figure 7F). We also extracted the scRNA-seq data on human metastatic TNBC cells to the brain from another dataset. Two batches of data were integrated, and QC was performed to remove the low-quality cells (Supplementary Figures 8A,B). Retained cells were clustered (Supplementary Figure 8C), and major cell types were defined (Supplementary Figures 8D,E). Cancer cells were then subset for further analysis (Supplementary Figure 8F).

**FIGURE 3:**
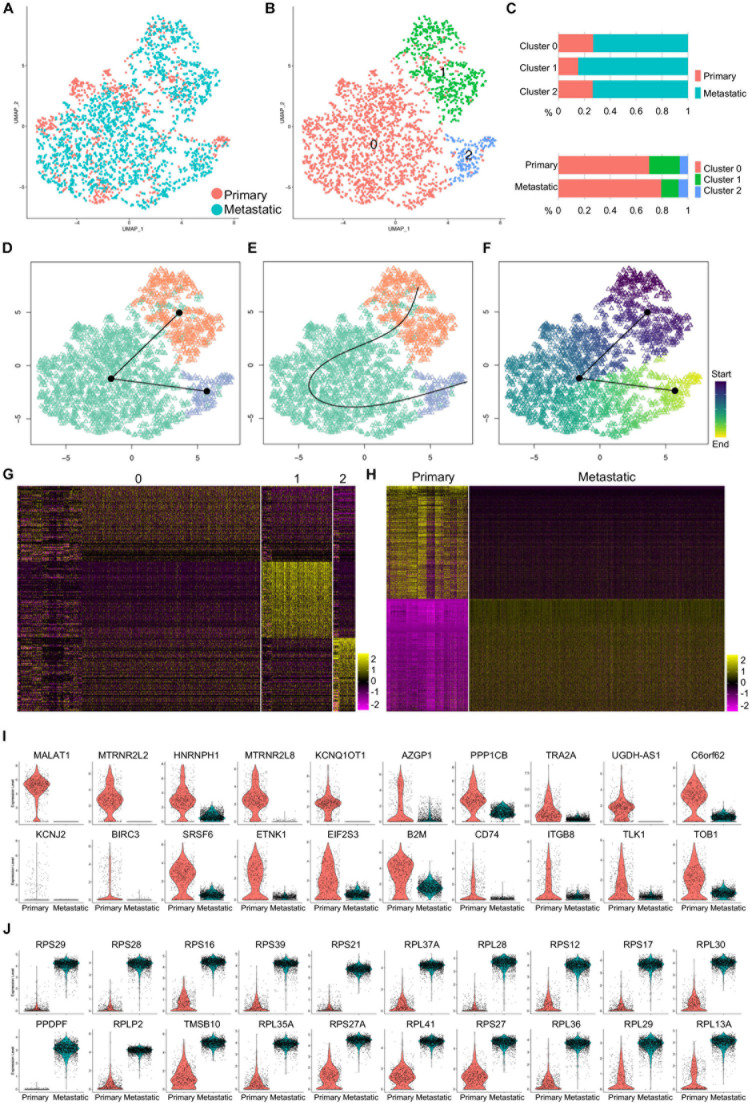
Gene expression patterns of primary and metastatic cancer cells of human TNBC cells. **(A)** Purified primary and metastatic cancer cells were integrated, and UMAP showed cell distribution. **(B)** Cells were clustered, and three clusters were identified. **(C)** The cell percentage of primary and metastatic cancer cells in each cluster and the cell percentage of each cluster in primary and metastatic cancer cells were quantified. **(D,E)** Slingshot analysis identified the inferred trajectory **(D)** and principal curves **(E)** of the cancer cell clusters. **(F)** Pseudotime analysis for the cancer cell clusters was visualized by UMAP. **(G,H)** Heatmap visualization of the top 100 genes of each cancer cell cluster **(G)** and top 500 genes of the primary and metastatic cancer cells **(H)**. **(I,J)** The violin plot showed the top 20 genes upregulated in primary **(I)** or metastatic **(J)** cancer cells.

The purified primary and metastatic cancer cells were integrated and clustered (Figure 3A), and three major cell clusters were identified (Figure 3B). Quantitatively, primary cancer cells shared equal percentage in cluster 0 and cluster 2 and had lower percentage in cluster 1 (Figure 3C). To determine the lineage differentiation potentials of the cancer clusters, we loaded the cells into Slingshot packages, packages that provided unified interface to dozens of different trajectory inference methods *via* docker containers, for pseudotime analysis. The cancer cell clusters were matched to the clusters defined above, and the development went along a cluster 1–cluster 0–cluster 2 axis (Figure 3D). Analysis on inferred trajectory and principal curves demonstrated smoothed representations of each subcluster (Figure 3E). Slingshot analysis revealed a pseudotime starting from cluster 1 and ending at cluster 2 (Figure 3F), which is consistent with the quantification analysis that primary cells contributed higher-percentage cells to cluster 1 and lower-percentage cells to cluster 2 compared to metastatic cells (Figure 3C, bottom panel). This analysis suggested that most metastatic cells (cluster 2) were mostly derived from less metastatic cells (clusters 0 and 1).

Comparative analysis revealed gene expression patterns of the three clusters, which was presented by the top 100 genes of each cluster (Figure 3G). Gene profiles visualized by the top 500 genes confirmed the specific gene expression patterns of both primary and metastatic cancer cells (Figure 3H). We then visualized the top 20 upregulated genes of both primary and metastatic cancer cells and showed their expression by violin plots (Figures 3I,J). Many of the genes upregulated in primary cancer cells showed some potential association in breast cancer progression and metastasis inhibition. In both genetically engineered mouse models and xenograft models, lncRNA MALAT1 overexpression inhibits, while MALAT1 deficiency induces breast cancer metastasis, which is reversed by re-expression of MALAT1, suggesting that MALAT1 is a metastasis-suppressing lncRNA (Kim et al., 2018). SRSF3 and HNRNPH1 regulate a splicing hotspot of HER2 in breast cancer cells (Gautrey et al., 2015). Another lncRNA KCNQ1OT1 is correlated with human breast cancer cell development through inverse regulation of miR-145 (Feng et al., 2018). TRA2A promotes paclitaxel resistance and tumor progression in TNBC *via* regulating alternative splicing (Liu et al., 2017). Notably, majority of the genes upregulated in metastatic cancer cells were ribosome protein genes (Figure 3J), suggesting that these genes were closely related with TNBC cell metastasis.

### Upregulation of RPL27A in Cancer Cells of Human TNBC Tissues

**FIGURE 4:**
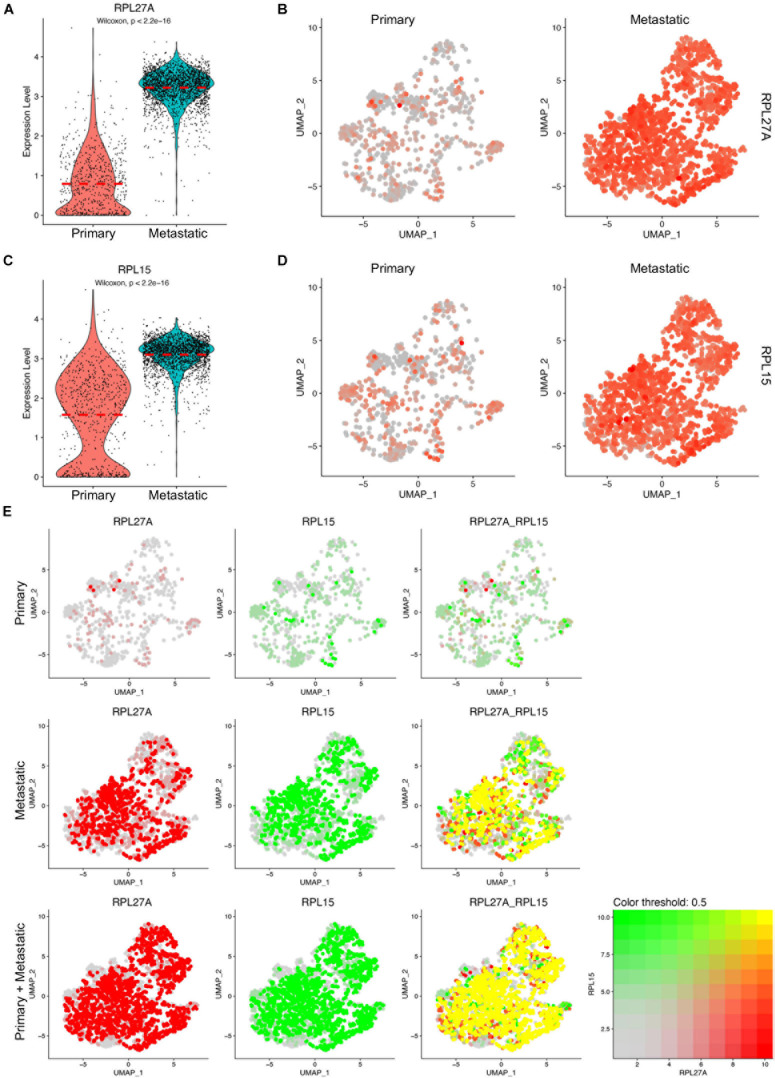
Upregulated RPL27A and RPL15 in metastatic TNBC cancer cells. **(A,C)** Violin plot representing the expression of RPL27A **(A)** and RPL15 **(C)** in primary and metastatic TNBC cancer cells. **(B,D)** UMAP confirmation of RPL27A **(B)** and RPL15 **(D)** expression in primary and metastatic TNBC cancer cells. **(E)** Colocalization of RPL27A and RPL15 transcription in the integrated TNBC cancer cells, primary TNBC cancer cells, and metastatic TNBC cancer cells.

Among the ribosome protein genes, the RPL27A and RPL15 genes were upregulated in metastatic cancer cells with significant differences, which was consistent with the data in mouse breast cancer models (Figures 4A,C). The split UMAP visualization of RPL27A and RPL15 expressions in primary and metastatic cancer cells confirmed these differences (Figures 4B,D). As RPL15 was a reported pro-metastasis gene in breast cancer (Ebright et al., 2020), we next checked the transcript colocalization of RPL27A with RPL15 in primary, metastatic, and integrated data. Surprisingly, RPL27A was well colocalized with RPL15 only in metastatic cancer cells; however, in primary cancer cells, the transcripts of the two genes were low and rarely colocalized (Figure 4E). Although Rpl29 and Rpl13a were downregulated in cancer cells of mouse models compared to normal mammary epithelial cells, these two genes were significantly upregulated in metastatic cancer cells compared to primary cancer cells (Supplementary Figure 9). These inconsistent findings suggested diverse functions of these genes or an inconsistent phenotype between mouse models and human tumor data (Supplementary Figure 9). Notably, the transcript levels of these four genes in total cells were also upregulated in metastatic TNBC tissues (Supplementary Figure 10A).

### Blocking RPL27A Blunted TNBC Cell Migration and Invasion

**FIGURE 5:**
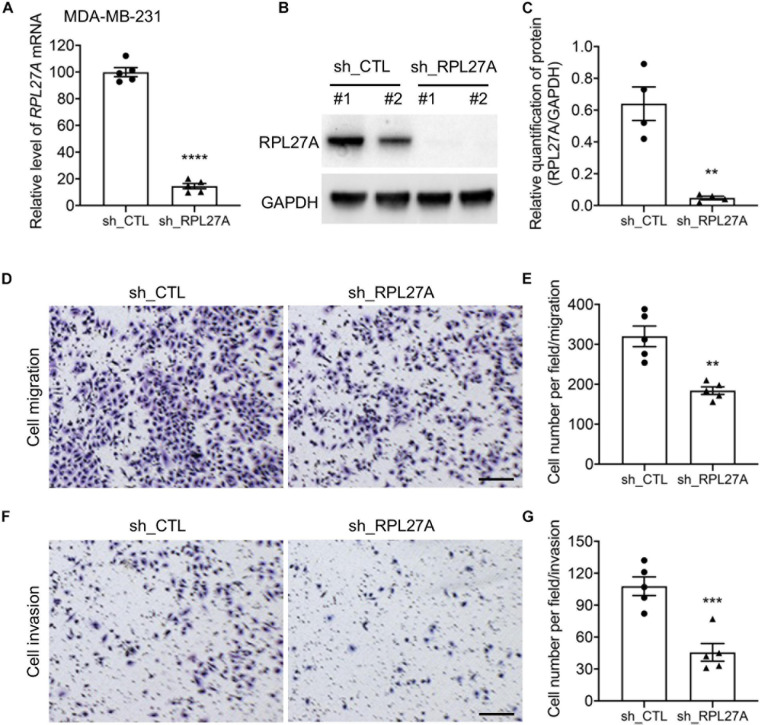
RPL27A knockdown decreased cell migration and invasion in the TNBC cell line. **(A–C)** Knockdown efficiency of RPL27A in MDA-MB-231 cells was confirmed by qRT-PCR **(A)** and western blot **(B,C)**. **(D,F)** Representative images of migration **(D)** and invasion **(F)** assays on RPL27A-knockdown and controls cells. **(E,G)** Quantification of cell numbers in each image of migration **(E)** and invasion **(G)** assays. Scale bar, 50 μm. **p < 0.01; ***p < 0.001.

To further confirm the potentials of RPL27A in regulating cancer cell metastasis, an *in vitro* model was generated on a human TNBC cell line, MDA-MB-231. RPL27A knockdown was performed on MDA-MB-231 cells by shRPL27A adenovirus transfection, and the knockdown efficiency was confirmed by quantitative real-time PCR (qRT-PCR) and western blot (Figures 5A–C). The cells were then used for migration and invasion assays, and significantly reduced cell numbers in both migration and invasion assays were confirmed by cell quantification (Figures 5D–G). These data further suggested that RPL27A was required for TNBC cell metastasis. More interestingly, RPL27A knockdown also caused significant downregulation of many other ribosomal genes (Supplementary Figure 10B).

### Deregulated Ribosomal Gene Expression in Metastatic TNBC Cells

**FIGURE 6:**
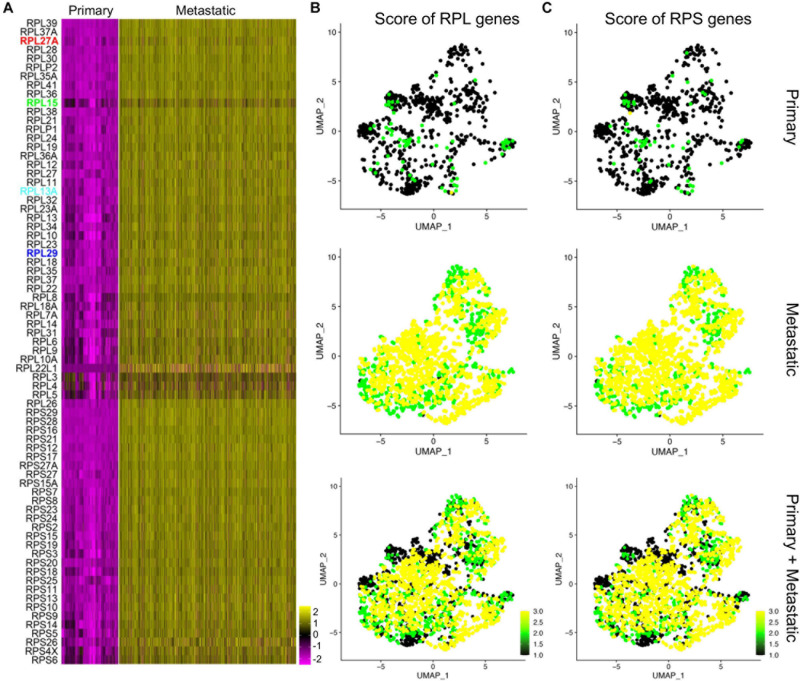
Deregulated ribosome protein genes in metastatic cancer cells. **(A)** Heatmap visualization of the expression of upregulated ribosome protein genes in the primary and metastatic cancer cells. **(B,C)** Scores of the RPL genes **(B)** and RPS genes **(C)** in primary, metastatic, and integrated data.

As many of the ribosomal protein genes were in the top gene list of the metastatic human TNBC cells, we proposed that the ribosomal protein genes were deregulated in TNBC metastasis. We then listed all the significantly differentially expressed ribosome protein genes and visualized them by heatmap (Figure 6A), and among them RPL27A, RPL15, RPL13A, and RPL29 were the top genes. We also separated the RPL genes and RPS genes and scored their expression by UMAPs (Figures 6B,C). Both the scores of RPL genes and RPS genes were significantly higher in metastatic cancer cell compared to those of primary cancer cells (Figures 6B,C). We next input these genes and their corresponding expression fold changes and *p*-values into IPA, and the top ingenuity canonical pathways and upstream regulators were visualized by bar plots (Supplementary Figures 10C,D). The graphic regulatory network based on the IPA revealed that EIF2 signaling was the core regulating signaling pathway (Supplementary Figure 10E).

### Activated EIF2 Signaling in Metastatic TNBC Cells

**FIGURE 7:**
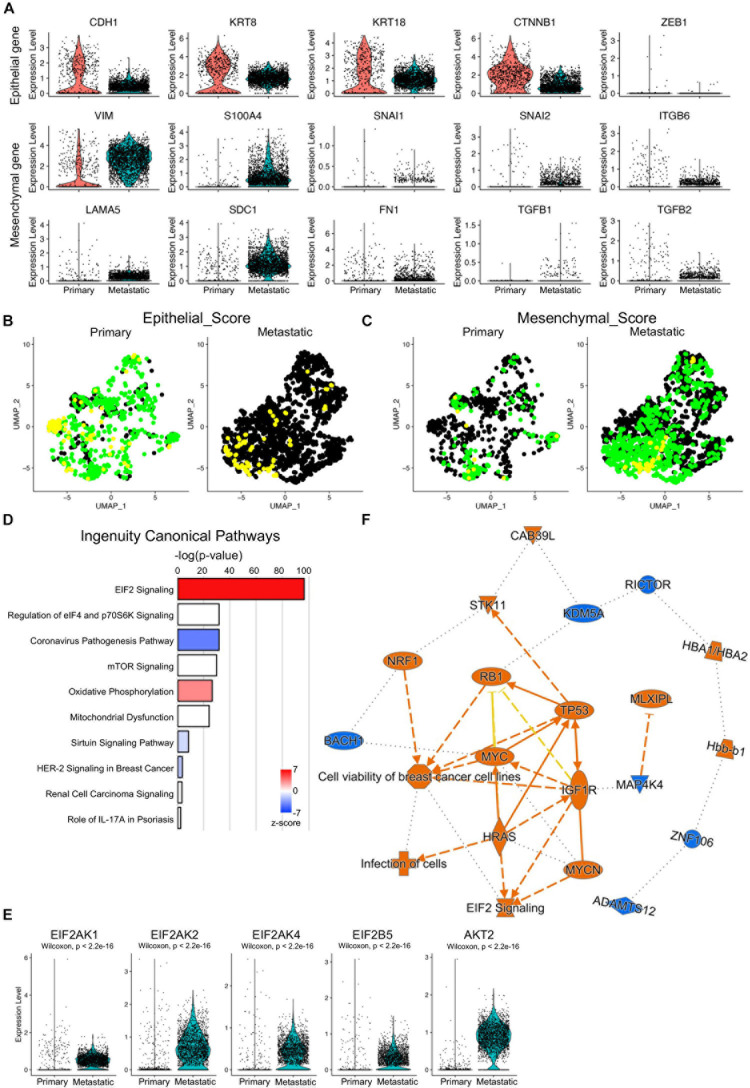
Increased EMT activities and activated EIF2 signaling in metastatic cancer cells. **(A)** Violin plot visualization of representative EMT marker gene expression in primary and metastatic cancer cells. **(B,C)** UMAP visualization of epithelial **(B)** and mesenchymal **(C)** scores of the primary and metastatic cancer cells. **(D)** Bar plots of the top ingenuity canonical pathways based on the upregulated genes of the metastatic cancer cells. **(E)** Increased expression of EIF2 signaling effector genes in metastatic cancer cells by violin plots. Color codes: orange, upregulated; blue, downregulated. Color shapes: different molecular/gene types. Lines: solid lines, direct regulation; dashed lines, indirect regulation. **(F)** Graphic regulatory networks of the top signaling pathways and regulators of metastatic cancer cells.

Epithelial–mesenchymal transition (EMT) is one of the major characteristics of breast cancer metastasis. EMT programs were believed to reflect a loss of epithelial gene expression signatures and morphologies that gave way to those associated with mesenchymal cells and their enhanced migratory and invasive behaviors (Gooding and Schiemann, 2020). To check the EMT activities of the primary and metastatic cancer cells, the transcriptional levels of canonical EMT-related signature genes, including epithelial feature genes (CDH1, KRT8, KRT18, CTNNB1, and ZEB1), mesenchymal feature genes (VIM, S100A4, FN1, and LAMA5), and EMT signature genes (SNAI1, SNAI2, ITGB6, TGFB1, and TGFB2) were visualized and compared between primary and metastatic cancer cells (Figure 7A). Significantly decreased epithelial signatures and increased mesenchymal and EMT signatures were found in metastatic cancer cells. Scoring of the epithelial signatures and mesenchymal signatures on primary and metastatic cancer cells further confirmed decreased epithelial scores and increased mesenchymal scores in metastatic cancer cells (Figures 7B,C).

To determine the major cancer metastasis-related signaling pathways, the differentially expressed genes between primary and metastatic cancer cells were input into IPA. The top 1 ingenuity canonical pathway was EIF2 signaling, and this (Figure 7D), together with the IPA on ribosome protein genes, further confirmed that EIF2 signaling was the key driver of the TNBC metastasis in human. To further confirm that, we checked the expression of the major component genes of EIF2 signaling and found upregulation of multiple genes, including EIF2AK1 (HRI protein coding gene), EIF2AK2 (PKR protein coding gene), EIF2AK4 (GCN2 protein coding gene), EIF2B5, and AKT2 (Figure 7E; Rios-Fuller et al., 2020). Other ingenuity canonical pathways included regulation of eIF4 and p70S6K signaling and mTOR signaling (Figure 7D), both of which were closely associated with TNBC biological activities (Madden et al., 2014; Wang et al., 2017; Zagorac et al., 2018). Graphic regulatory networks integrating ingenuity canonical pathways and upstream regulators of the metastatic cells gave a core pathway of cell viability of breast cancer cells, which was closely related with EIF2 signaling and regulators of RB1, TP53, and MYC (Figure 7F).

**FIGURE 8:**
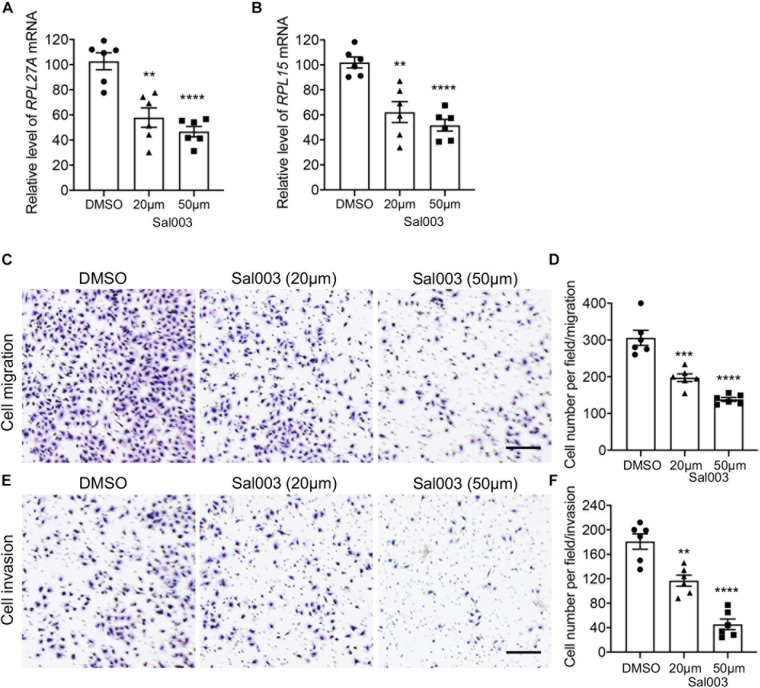
Blocking EIF2 signaling decreased TNBC cell migration and invasion. **(A,B)** mRNA levels of RPL27A and RPL15 in MDA-MB-231 cells after treatment of different doses of the EIF2 signaling inhibitor Sal003. **(C,E)** Representative images of migration **(C)** and invasion **(E)** assays on MDA-MB-231 cells after treatment of different doses of Sal003 or DMSO control. **(D,F)** Quantification of cell numbers in each image of migration **(D)** and invasion **(F)** assays. Scale bar, 50 μm. **p < 0.01; ***p < 0.001; ****p < 0.0001.

The salubrinal derivative Sal003 is the most commonly used eIF2α inhibitor II (Robert et al., 2006; Baltzis et al., 2007). To better validate the functions of EIF2 in TNBC metastasis, we inhibited EIF2 signaling in the MDA-MB-231 cells with Sal003 at different doses. Consistently, both the mRNA levels of RPL27A and RPL15 were significantly decreased after Sal003 treatment at a dose-dependent manner (Figures 8A,B), and this is also true for many other ribosome-related genes we detected (Supplementary Figure 10F). More importantly, Sal003 treatment dramatically attenuated MDA-MB-231 cell migration and invasion (Figures 8C–F), suggesting an attractively reduced metastasis potential in cancer cells when blocking EIF2 signaling. All these findings supplied basic foundations for further studies and potential therapeutic target development of TNBC and were likely to attract a broad audience.

## Discussion

Triple-negative breast cancer accounts for approximately 10–15% of all breast cancers and has a higher incidence in some specific ethnicities, including Latin, African, and African American women (Lund et al., 2009). These tumors are characterized by aggressive behaviors, with a trend to early relapse and capable of metastatically spreading to the lung, liver, and central nervous system, as well as having poorer survival (Dent et al., 2007). However, to date, targeted therapies have not significantly improved survival in patients with TNBC, and chemotherapy remains the standard of clinical care (Garrido-Castro et al., 2019). The identification of biomarkers that can help guide treatment decisions in TNBC remains a clinically unmet need.

To improve the therapeutic benefit of TNBC treatment, numbers of agents have been under exploration of clinical studies, including immunotherapies and targeted therapies in the networks of tumor–stroma, DNA damage response, cell surface or intracellular receptors, and signaling pathways as well as cell surface markers for selective drug delivery and antibody–drug conjugates (Won and Spruck, 2020). Recent advances in new agents have been made for specific subgroups of patients with PD-L1^+^ tumors (Schutz et al., 2017) or gBRCAm tumors (Won and Spruck, 2020). However, only a small subgroup of those patients responds to immune checkpoint or PARP inhibitors, and some often develop resistance and relapse. In these complex tumor microenvironments, a specific therapeutic agent often shows variable responses, thus compromising the survival endpoints, especially in an unselected TNBC population. Therefore, developing novel predictive biomarkers is crucial for selecting patients who will benefit the most from a given therapy.

The development of single-cell technologies provides additional insights on tumor microenvironments and diverse cell-type interactions and thus facilitates a compelling rationale for new treatments based on novel biomarkers (Ding et al., 2020; Fan et al., 2020; Guruprasad et al., 2021). Single-cell omics studies enable gene expression profiling at the single-cell levels and detection of gene expression differences within cell populations and among disease stages. Therefore, novel biomarkers related to cancer progression and response to target therapies might be revealed by these novel technologies. Here, in the current study, by using scRNA-seq profiling of cancer-associated cells and comparative analysis, we developed specific gene expression patterns during TNBC development, progression, and metastasis in both murine and human cells. Among the deregulated gene candidates, ribosome protein genes were highly involved in TNBC development and metastasis, and two of the most consistent genes between mouse models and human cancer were RPL15 and RPL27A.

A most recent study conducted an *in vivo* genome-wide CRISPR activation screen in circulating tumor cells from breast cancer patients to identify genes that promoted distant metastasis in mice. Genes coding for ribosomal proteins and regulators of translation were enriched in this screen. Overexpression of RPL15 increased metastatic growth in multiple organs and selectively enhanced translation of other ribosomal proteins and cell cycle regulators (Ebright et al., 2020). This report, in coordination with our data, confirmed the participation of RPL15 in breast cancer cell metastasis.

RPL27A, a ribosomal subunit protein, has been well studied to conduct diverse responsibilities in multiple disease phenotypes; however, its functions in cancer, especially in breast cancers, have been rarely determined with a few exceptions. RPL27A was one of the genes that are frequently co-expressed in breast cancer patients with RPS16, which was thought to be one of the targets of miR-7641 (Reza et al., 2017). RPL27A was also one of the differentially expressed genes in the ribosome pathway involved in breast cancer using the Gibbs sampling approach (Zhou et al., 2017). Moreover, RPL27A was thought to be a target of miR-595 and might contribute to the myelodysplastic phenotype through ribosomal dysgenesis *via* the effects of p53 activation, ribosome synthesis, and maturation (Alkhatabi et al., 2016). Although these studies had some correlation with RPL27A, the specific functions and mechanisms of RPL27A were largely unknown. In the current study, we well described the involvement of RPL27A in TNBC development and metastasis in murine and human cells. Consistent to its upregulation in metastatic cancer cells, knockdown of RPL27A significantly decreased the migration and invasion of the TNBC cell line, as well as other ribosome-related genes. IPA identified a key signaling pathway, EIF2 signaling, which was potentially driving the deregulation of ribosomal genes and metastasis of the TNBC cancer cells. Although the involvements of EIF2 in initiation and progression of tumor have been actively studied, the current understanding of the roles eIF2α in cancer (Holcik, 2015; Koromilas, 2015; Hao et al., 2020), especially breast cancer, is still unclear and needs further investigation (Zheng et al., 2014). Our *in vitro* assays demonstrated that blocking EIF2 signaling significantly inhibited TNBC cell migration and invasion. Although the mechanisms of this signaling pathway in breast cancer metastasis needed further investigation, our findings shed light on the discovery of novel biomarkers for TNBC and might generate a foundation for future studies of ribosome proteins in the development, progression, and metastasis of TNBC. We believed that RPL27A was a specific biomarker for TNBC diagnosis and was potentially a novel therapeutic target for TNBC.

## Data Availability Statement

The datasets presented in this study can be found in online repositories. The names of the repository/repositories and accession number(s) can be found in the article/Supplementary Material.

## Ethics Statement

All human lung experiments were approved by the Institutional Review Board of Tianjin Medical University Cancer Institute and Hospital and were in accordance with the guidelines outlined by the IRB. The patients/participants provided their written informed consent to participate in this study.

## Author Contributions

WZ and ZT conceived the study and prepared the manuscript. WZ and XL analyzed the data. WZ and YZ prepared the reagents and performed the experiments. JW, XW, and LS reviewed the result and the discussion of the manuscript. All authors read and approved the manuscript.

## Conflict of Interest

The authors declare that the research was conducted in the absence of any commercial or financial relationships that could be construed as a potential conflict of interest.

## Publisher’s Note

All claims expressed in this article are solely those of the authors and do not necessarily represent those of their affiliated organizations, or those of the publisher, the editors and the reviewers. Any product that may be evaluated in this article, or claim that may be made by its manufacturer, is not guaranteed or endorsed by the publisher.

## Abbreviations

scRNA-seq: single-cell RNA sequencing
TNBC: triple-negative breast cancer
RPL27A: ribosomal protein L27a
RPL15: ribosomal protein L15
RPL29: ribosomal protein L29
RPL13A: ribosomal protein L13a
UMAP: Uniform Manifold Approximation and Projection
ER: estrogen receptor
PR: progesterone receptor
GEO: Gene Expression Omnibus
SRA: Sequence Read Archive
EMT: epithelial–mesenchymal transition
EIF2: eukaryotic initiation factor 2.

## References

[B1] Alkhatabi H. A. McLornan D. P. Kulasekararaj A. G. Malik F. Seidl T. Darling D. (2016). RPL27A is a target of miR-595 and may contribute to the myelodysplastic phenotype through ribosomal dysgenesis. *Oncotarget* 7 47875–47890. 10.18632/oncotarget.10293 27374104 PMC5216985

[B2] Bach K. Pensa S. Grzelak M. Hadfield J. Adams D. J. Marioni J. C. (2017). Differentiation dynamics of mammary epithelial cells revealed by single-cell RNA sequencing. *Nat. Commun.* 8:2128. 10.1038/s41467-017-02001-5 29225342 PMC5723634

[B3] Baltzis D. Pluquet O. Papadakis A. I. Kazemi S. Qu L. K. Koromilas A. E. (2007). The eIF2alpha kinases PERK and PKR activate glycogen synthase kinase 3 to promote the proteasomal degradation of p53. *J. Biol. Chem.* 282 31675–31687. 10.1074/jbc.M704491200 17785458

[B4] Bianchini G. Balko J. M. Mayer I. A. Sanders M. E. Gianni L. (2016). Triple-negative breast cancer: challenges and opportunities of a heterogeneous disease. *Nat. Rev. Clin. Oncol.* 13 674–690. 10.1038/nrclinonc.2016.66 27184417 PMC5461122

[B5] Bresson L. Faraldo M. M. Di-Cicco A. Quintanilla M. Glukhova M. A. Deugnier M. A. (2018). Podoplanin regulates mammary stem cell function and tumorigenesis by potentiating Wnt/beta-catenin signaling. *Development* 145:dev160382. 10.1242/dev.160382 29361573

[B6] Ciriello G. Gatza M. L. Beck A. H. Wilkerson M. D. Rhie S. K. Pastore A. (2015). Comprehensive molecular portraits of invasive lobular breast cancer. *Cell* 163 506–519. 10.1016/j.cell.2015.09.033 26451490 PMC4603750

[B7] Collignon J. Lousberg L. Schroeder H. Jerusalem G. (2016). Triple-negative breast cancer: treatment challenges and solutions. *Breast Cancer (Dove Med. Press)* 8 93–107. 10.2147/BCTT.S69488 27284266 PMC4881925

[B8] Dave B. Granados-Principal S. Zhu R. Benz S. Rabizadeh S. Soon-Shiong P. (2014). Targeting RPL39 and MLF2 reduces tumor initiation and metastasis in breast cancer by inhibiting nitric oxide synthase signaling. *Proc. Natl. Acad. Sci. U.S.A.* 111 8838–8843. 10.1073/pnas.1320769111 24876273 PMC4066479

[B9] Dent R. Trudeau M. Pritchard K. I. Hanna W. M. Kahn H. K. Sawka C. A. (2007). Triple-negative breast cancer: clinical features and patterns of recurrence. *Clin. Cancer Res.* 13(15 Pt 1) 4429–4434. 10.1158/1078-0432.CCR-06-3045 17671126

[B10] Ding S. Chen X. Shen K. (2020). Single-cell RNA sequencing in breast cancer: understanding tumor heterogeneity and paving roads to individualized therapy. *Cancer Commun. (Lond)* 40 329–344. 10.1002/cac2.12078 32654419 PMC7427308

[B11] Ebright R. Y. Lee S. Wittner B. S. Niederhoffer K. L. Nicholson B. T. Bardia A. (2020). Deregulation of ribosomal protein expression and translation promotes breast cancer metastasis. *Science* 367 1468–1473. 10.1126/science.aay0939 32029688 PMC7307008

[B12] Fan J. Slowikowski K. Zhang F. (2020). Single-cell transcriptomics in cancer: computational challenges and opportunities. *Exp. Mol. Med.* 52 1452–1465. 10.1038/s12276-020-0422-0 32929226 PMC8080633

[B13] Fancello L. Kampen K. R. Hofman I. J. Verbeeck J. De Keersmaecker K. (2017). The ribosomal protein gene RPL5 is a *haploinsufficient tumor* suppressor in multiple cancer types. *Oncotarget* 8 14462–14478. 10.18632/oncotarget.14895 28147343 PMC5362418

[B14] Feng W. Wang C. Liang C. Yang H. Chen D. Yu X. (2018). The dysregulated expression of KCNQ1OT1 and its interaction with downstream factors miR-145/CCNE2 in breast cancer cells. *Cell Physiol. Biochem.* 49 432–446. 10.1159/000492978 30157476

[B15] Fu D. He C. Wei J. Zhang Z. Luo Y. Tan H. (2018). PGK1 is a potential survival biomarker and invasion promoter by regulating the HIF-1alpha-mediated epithelial-mesenchymal transition process in breast cancer. *Cell Physiol. Biochem.* 51 2434–2444. 10.1159/000495900 30537744

[B16] Garrido-Castro A. C. Lin N. U. Polyak K. (2019). Insights into molecular classifications of triple-negative breast cancer: improving patient selection for treatment. *Cancer Discov.* 9 176–198. 10.1158/2159-8290.CD-18-1177 30679171 PMC6387871

[B17] Gautrey H. Jackson C. Dittrich A. L. Browell D. Lennard T. Tyson-Capper A. (2015). SRSF3 and hnRNP H1 regulate a splicing hotspot of HER2 in breast cancer cells. *RNA Biol.* 12 1139–1151. 10.1080/15476286.2015.1076610 26367347 PMC4829299

[B18] Gooding A. J. Schiemann W. P. (2020). Epithelial-Mesenchymal transition programs and cancer stem cell phenotypes: mediators of breast cancer therapy resistance. *Mol. Cancer Res.* 18 1257–1270. 10.1158/1541-7786.MCR-20-0067 32503922 PMC7483945

[B19] Gothlin Eremo A. Lagergren K. Othman L. Montgomery S. Andersson G. Tina E. (2020). Evaluation of SPP1/osteopontin expression as predictor of recurrence in tamoxifen treated breast cancer. *Sci. Rep.* 10:1451. 10.1038/s41598-020-58323-w 31996744 PMC6989629

[B20] Guruprasad P. Lee Y. G. Kim K. H. Ruella M. (2021). The current landscape of single-cell transcriptomics for cancer immunotherapy. *J. Exp. Med.* 218:e20201574. 10.1084/jem.20201574 33601414 PMC7754680

[B21] Hao P. Q. Yu J. J. Ward R. Liu Y. Hao Q. An S. (2020). Eukaryotic translation initiation factors as promising targets in cancer therapy. *Cell Commun. Signal.* 18:175. 10.1186/s12964-020-00607-9 33148274 PMC7640403

[B22] Holcik M. (2015). Could the elF2 alpha-independent translation be the achilles heel of cancer? *Front. Oncol.* 5:264. 10.3389/fonc.2015.00264 26636041 PMC4659918

[B23] Hong M. Kim H. Kim I. (2014). Ribosomal protein L19 overexpression activates the unfolded protein response and sensitizes MCF7 breast cancer cells to endoplasmic reticulum stress-induced cell death. *Biochem. Biophys. Res. Commun.* 450 673–678. 10.1016/j.bbrc.2014.06.036 24950402

[B24] Jogi A. Brennan D. J. Ryden L. Magnusson K. Ferno M. Stal O. (2009). Nuclear expression of the RNA-binding protein RBM3 is associated with an improved clinical outcome in breast cancer. *Mod. Pathol.* 22 1564–1574. 10.1038/modpathol.2009.124 19734850

[B25] Karaayvaz M. Cristea S. Gillespie S. M. Patel A. P. Mylvaganam R. Luo C. C. (2018). Unravelling subclonal heterogeneity and aggressive disease states in TNBC through single-cell RNA-seq. *Nat. Commun.* 9:3588. 10.1038/s41467-018-06052-0 30181541 PMC6123496

[B26] Kendrick H. Regan J. L. Magnay F. A. Grigoriadis A. Mitsopoulos C. Zvelebil M. (2008). Transcriptome analysis of mammary epithelial subpopulations identifies novel determinants of lineage commitment and cell fate. *BMC Genomics* 9:591. 10.1186/1471-2164-9-591 19063729 PMC2629782

[B27] Kim J. Piao H. L. Kim B. J. Yao F. Han Z. Wang Y. (2018). Long noncoding RNA MALAT1 suppresses breast cancer metastasis. *Nat. Genet.* 50 1705–1715. 10.1038/s41588-018-0252-3 30349115 PMC6265076

[B28] Kioulafa M. Kaklamanis L. Stathopoulos E. Mavroudis D. Georgoulias V. Lianidou E. S. (2009). Kallikrein 10 (KLK10) methylation as a novel prognostic biomarker in early breast cancer. *Ann. Oncol.* 20 1020–1025. 10.1093/annonc/mdn733 19150938

[B29] Koromilas A. E. (2015). Roles of the translation initiation factor eIF2 alpha serine 51 phosphorylation in cancer formation and treatment. *Bba-Gene Regul. Mech.* 1849 871–880. 10.1016/j.bbagrm.2014.12.007 25497381

[B30] Lee S. J. Chae Y. S. Kim J. G. Kim W. W. Jung J. H. Park H. Y. (2014). AQP5 expression predicts survival in patients with early breast cancer. *Ann. Surg. Oncol.* 21 375–383. 10.1245/s10434-013-3317-7 24114055

[B31] Liu T. Sun H. Zhu D. Dong X. Liu F. Liang X. (2017). TRA2A promoted paclitaxel resistance and tumor progression in triple-negative breast cancers via regulating alternative splicing. *Mol. Cancer Ther.* 16 1377–1388. 10.1158/1535-7163.MCT-17-0026 28416606

[B32] Lund M. J. Trivers K. F. Porter P. L. Coates R. J. Leyland-Jones B. Brawley O. W. (2009). Race and triple negative threats to breast cancer survival: a population-based study in Atlanta, GA. *Breast Cancer Res. Treat.* 113 357–370. 10.1007/s10549-008-9926-3 18324472

[B33] Madden J. M. Mueller K. L. Bollig-Fischer A. Stemmer P. Mattingly R. R. Boerner J. L. (2014). Abrogating phosphorylation of eIF4B is required for EGFR and mTOR inhibitor synergy in triple-negative breast cancer. *Breast Cancer Res. Treat.* 147 283–293. 10.1007/s10549-014-3102-8 25129346 PMC4171954

[B34] Ostrakhovitch E. A. Li S. S. (2010). NIP1/DUOXA1 expression in epithelial breast cancer cells: regulation of cell adhesion and actin dynamics. *Breast Cancer Res. Treat.* 119 773–786. 10.1007/s10549-009-0372-7 19322654

[B35] Penzo M. Montanaro L. Trere D. Derenzini M. (2019). The ribosome biogenesis-cancer connection. *Cells* 8:55. 10.3390/cells8010055 30650663 PMC6356843

[B36] Reza A. Choi Y. J. Yuan Y. G. Das J. Yasuda H. Kim J. H. (2017). MicroRNA-7641 is a regulator of ribosomal proteins and a promising targeting factor to improve the efficacy of cancer therapy. *Sci. Rep.* 7:8365. 10.1038/s41598-017-08737-w 28827731 PMC5566380

[B37] Rios-Fuller T. J. Mahe M. Walters B. Abbadi D. Perez-Baos S. Gadi A. (2020). Translation regulation by eIF2alpha phosphorylation and mTORC1 signaling pathways in non-communicable diseases (n.d.). *Int. J. Mol. Sci.* 21:5301. 10.3390/ijms21155301 32722591 PMC7432514

[B38] Robert F. Kapp L. D. Khan S. N. Acker M. G. Kolitz S. Kazemi S. (2006). Initiation of protein synthesis by hepatitis C virus is refractory to reduced eIF2 center dot GTP center dot Met-tRNA(i)(Met) ternary complex availability. *Mol. Biol. Cell* 17 4632–4644. 10.1091/mbc.E06-06-0478 16928960 PMC1635388

[B39] Ruggero D. Pandolfi P. P. (2003). Does the ribosome translate cancer? *Nat. Rev. Cancer* 3 179–192. 10.1038/nrc1015 12612653

[B40] Schutz F. Stefanovic S. Mayer L. von Au A. Domschke C. Sohn C. (2017). PD-1/PD-L1 pathway in breast cancer. *Oncol. Res. Treat.* 40 294–297. 10.1159/000464353 28346916

[B41] Silvera D. Formenti S. C. Schneider R. J. (2010). Translational control in cancer. *Nat. Rev. Cancer* 10 254–266. 10.1038/nrc2824 20332778

[B42] Subramonian D. Raghunayakula S. Olsen J. V. Beningo K. A. Paschen W. Zhang X. D. (2014). Analysis of changes in SUMO-2/3 modification during breast cancer progression and metastasis. *J. Proteome Res.* 13 3905–3918. 10.1021/pr500119a 25072996

[B43] Tuohy V. K. (2014). Retired self-proteins as vaccine targets for primary immunoprevention of adult-onset cancers. *Expert. Rev. Vaccines* 13 1447–1462. 10.1586/14760584.2014.953063 25172043

[B44] Wang X. Yao J. Wang J. Zhang Q. Brady S. W. Arun B. (2017). Targeting Aberrant p70S6K activation for estrogen receptor-negative breast cancer prevention. *Cancer Prev. Res. (Phila)* 10 641–650. 10.1158/1940-6207.CAPR-17-0106 28877935 PMC5668174

[B45] Weymouth N. Shi Z. Rockey D. C. (2012). Smooth muscle alpha actin is specifically required for the maintenance of lactation. *Dev. Biol.* 363 1–14. 10.1016/j.ydbio.2011.11.002 22123032 PMC4151467

[B46] Won K. A. Spruck C. (2020). Triplenegative breast cancer therapy: current and future perspectives (Review). *Int. J. Oncol.* 57 1245–1261. 10.3892/ijo.2020.5135 33174058 PMC7646583

[B47] Yeo S. K. Zhu X. Okamoto T. Hao M. Wang C. Lu P. (2020). Single-cell RNA-sequencing reveals distinct patterns of cell state heterogeneity in mouse models of breast cancer. *Elife* 9:e58810. 10.7554/eLife.58810 32840210 PMC7447441

[B48] Yin L. Duan J. J. Bian X. W. Yu S. C. (2020). Triple-negative breast cancer molecular subtyping and treatment progress. *Breast Cancer Res.* 22:61. 10.1186/s13058-020-01296-5 32517735 PMC7285581

[B49] Zagorac I. Fernandez-Gaitero S. Penning R. Post H. Bueno M. J. Mouron S. (2018). *In vivo phosphoproteomics* reveals kinase activity profiles that predict treatment outcome in triple-negative breast cancer. *Nat. Commun.* 9:3501. 10.1038/s41467-018-05742-z 30158526 PMC6115463

[B50] Zhang D. Jin N. Sun W. Li X. Liu B. Xie Z. (2017). Phosphoglycerate mutase 1 promotes cancer cell migration independent of its metabolic activity. *Oncogene* 36 2900–2909. 10.1038/onc.2016.446 27991922

[B51] Zheng Q. L. Ye J. J. Cao J. (2014). Translational regulator eIF2 alpha in tumor. *Tumor. Biol.* 35 6255–6264. 10.1007/s13277-014-1789-0 24609900

[B52] Zhou G. Lu M. Q. Li D. J. Gao B. A. Guo R. (2017). Identification of differentially expressed molecular functions associated with breast cancer using Gibbs sampling. *Oncol. Lett.* 14 7489–7494. 10.3892/ol.2017.7158 29344193 PMC5755151

[B53] Zhou X. Liao W. J. Liao J. M. Liao P. Lu H. (2015). Ribosomal proteins: functions beyond the ribosome. *J. Mol. Cell Biol.* 7 92–104. 10.1093/jmcb/mjv014 25735597 PMC4481666

