# Supplementary material for: Ribosome Proteins Represented by RPL27A Mark the Development and Metastasis of Triple-Negative Breast Cancer in Mouse and Human
Source: Front Cell Dev Biol. 2021 Aug 23;9:716730. doi: 10.3389/fcell.2021.716730 (PMC8419227; doi:10.3389/fcell.2021.716730)
Supplement: Supplementary file 1 [file Data_Sheet_1.pdf]

Figure S1

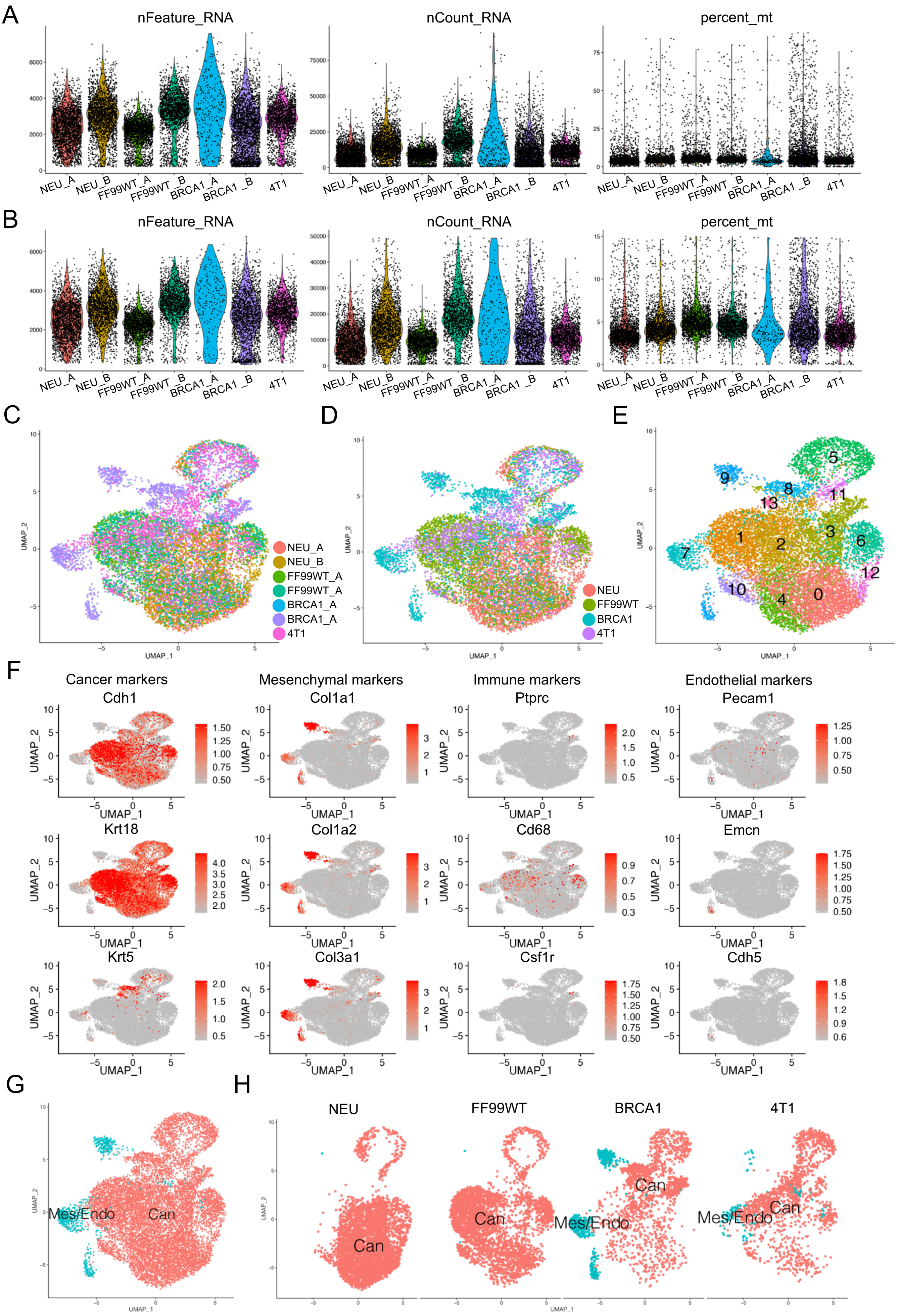

Supplementary Figure 1 | Quality control and cell type identification of scRNA-seq data on breast cancer mouse models. Cell qualities of each sample before (**A**) and after QC (**B**). Violin plots were used to visualize the total number of genes detected each cell (nFeature_RNA), number of transcripts each cell (nCount_RNA), and percentage of mitochondrial genes of each cell (percent.MT) of each scRNA-seq sample before (**A**) and after (**B**) QC. scRNA-seq data of four breast cancer mouse models were integrated and distribution of samples (**C**) and models (**D**) was visualized by UMAPs. (**E**) The integrated data were clustered and major clusters were identified. Cell type marker expression (**F**) and major cell type identification (**G**, **H**) were visualized by UMAPs.

Figure S2

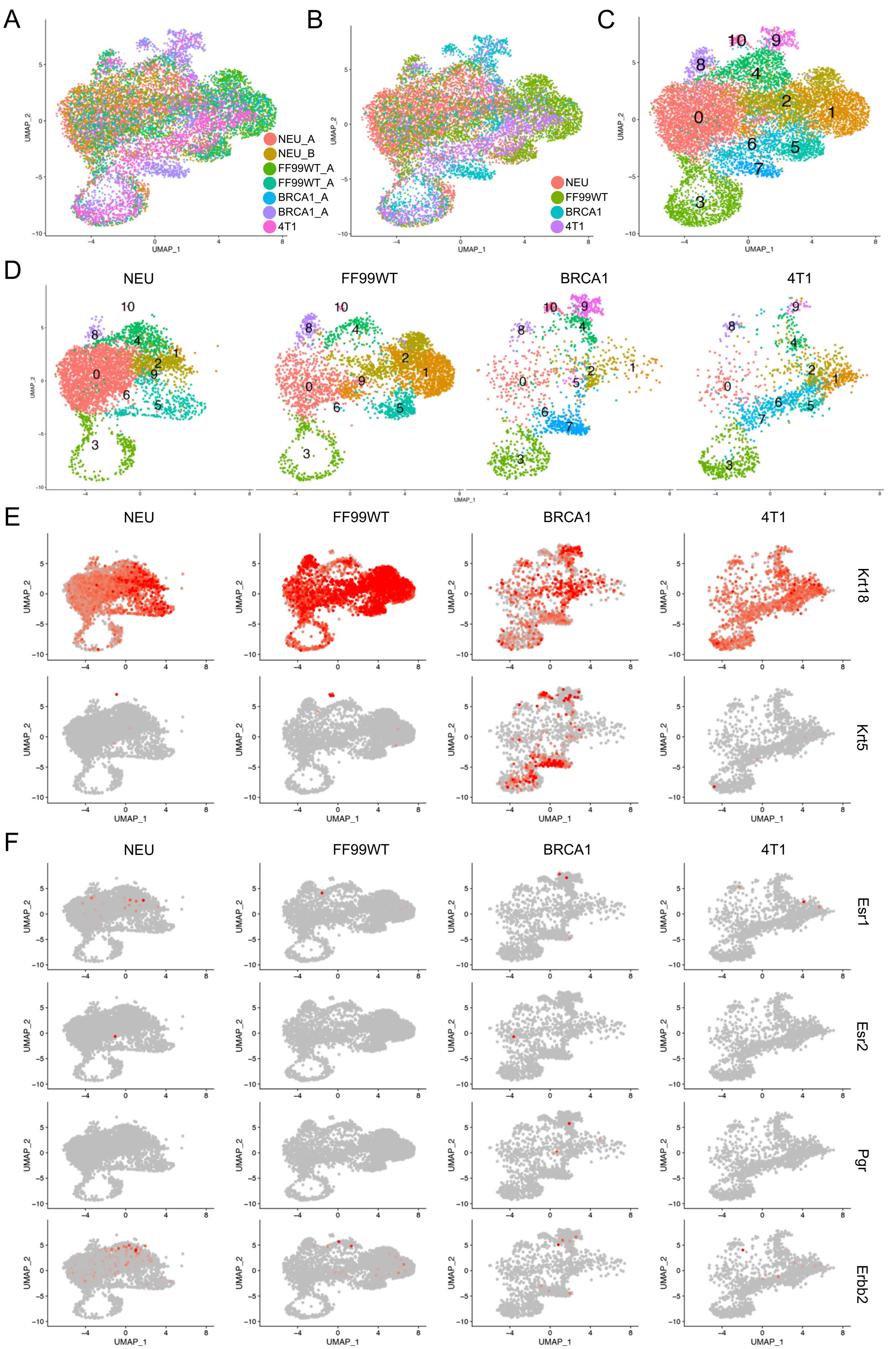

Supplementary Figure 2 | Cancer cell extraction from the scRNA-seq on breast cancer mouse models. (**A**-**B**) Cancer cells were extracted and cells from each sample (**A**) and each model (**B**) were visualized by UMAPs. (**C**-**D**) The cancer cells were clustered (**C**) and cluster distribution in each mouse model were visualized by split UMAPs (**D**). (**E**) The expression of luminal marker Krt18 and basal marker Krt5 in the cancer cells of each cancer model. (**F**) The transcripts of estrogen receptor genes, progesterone receptor gene and HER2 (Erbb2) gene in the cancer cells of each cancer model.

Figure S3

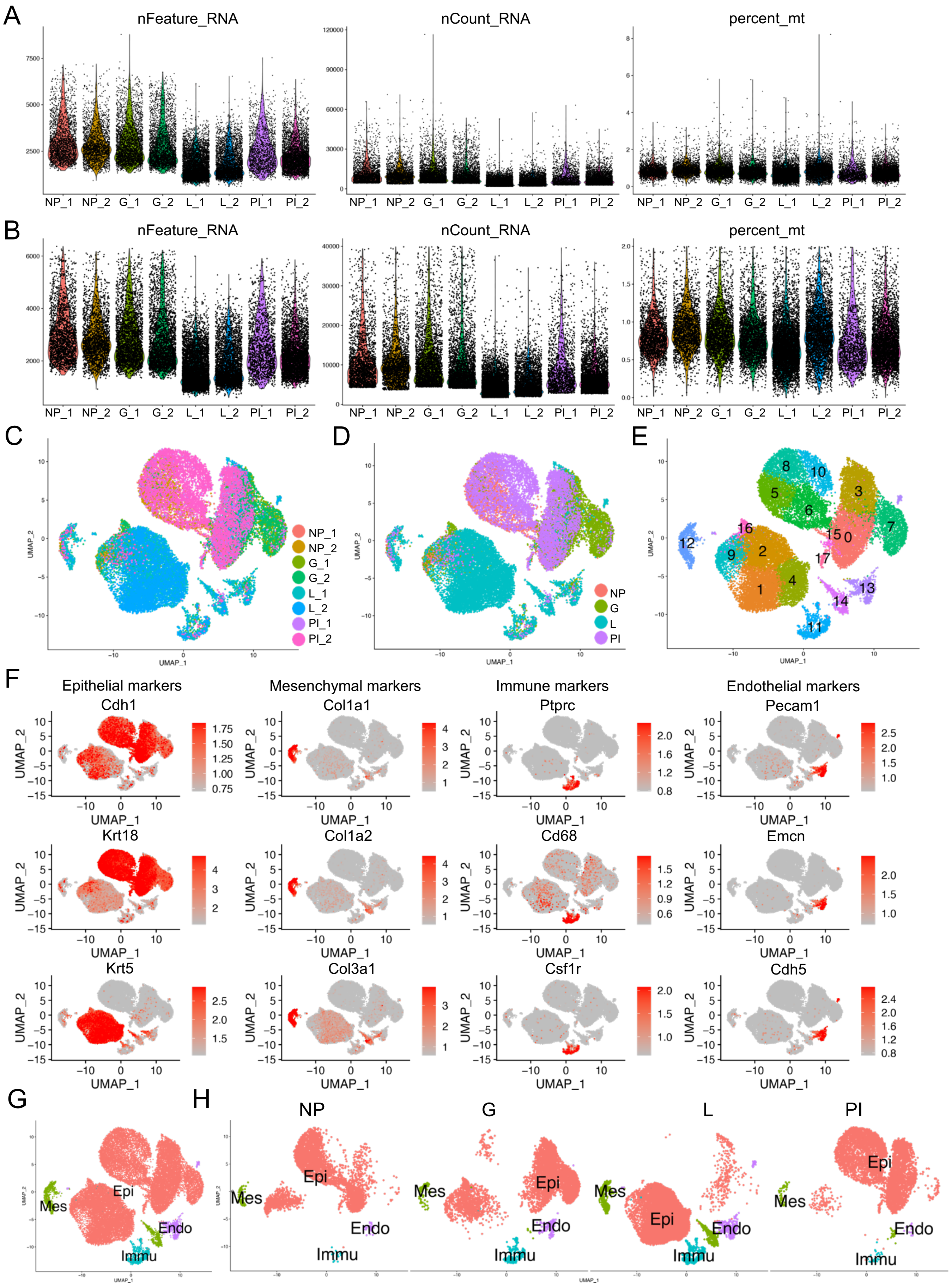

Supplementary Figure 3 | Quality control and cell type identification of scRNA-seq data on normal mouse mammary tissues. Cell qualities of each sample before (**A**) and after QC (**B**). Violin plots were used to visualize the total number of genes detected each cell (nFeature_RNA), number of transcripts each cell (nCount_RNA), and percentage of mitochondrial genes of each cell (percent.MT) of each scRNA-seq sample before (**A**) and after (**B**) QC. scRNA-seq data of mammary tissues of four stages were integrated and distribution of each sample (**C**) or each model (**D**) was visualized by UMAPs. (**E**) The integrated data were clustered and major clusters were identified. (**F**) Canonical cell type marker expression in the integrated data. Major cell type identified based on the gene expression (**F**) were visualized by UMAPs of the integrated data (**G**) or each developmental stage data (**H**).

Figure S4

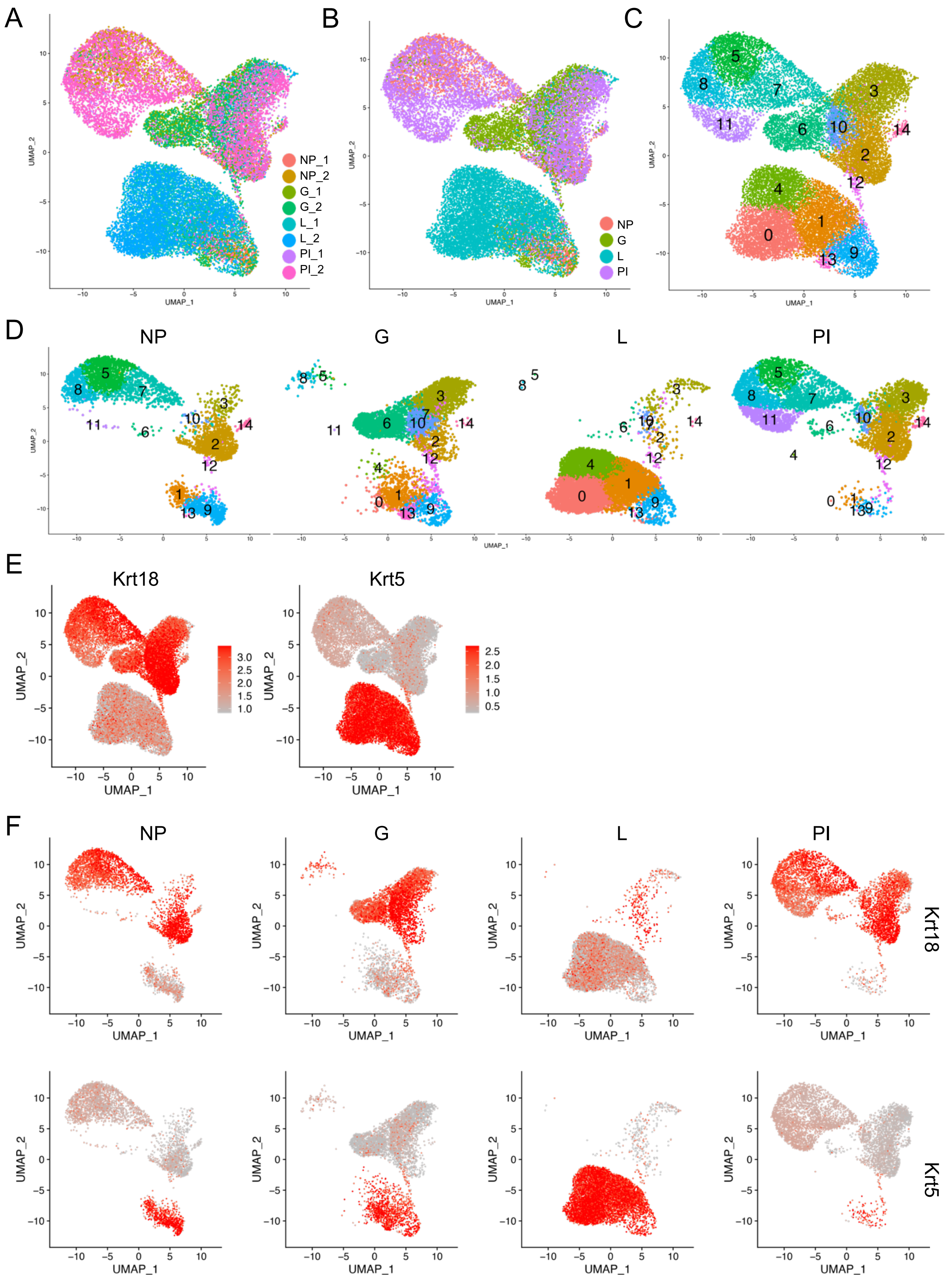

Supplementary Figure 4 | Epithelial cell extraction from the scRNA-seq on normal mouse mammary tissues. (**A**-**B**) Extracted epithelial cells visualized to display cell distribution from each sample (**A**) and each developmental stage (**B**). (**C**-**D**) The epithelial cell clusters of the integrated data and split data were visualized by UMAPs. (**E**-**F**) The expression of luminal marker Krt18 and basal marker Krt5 in the epithelial cells of the integrated data (**E**) and different developmental stages (**F**).

Figure S5

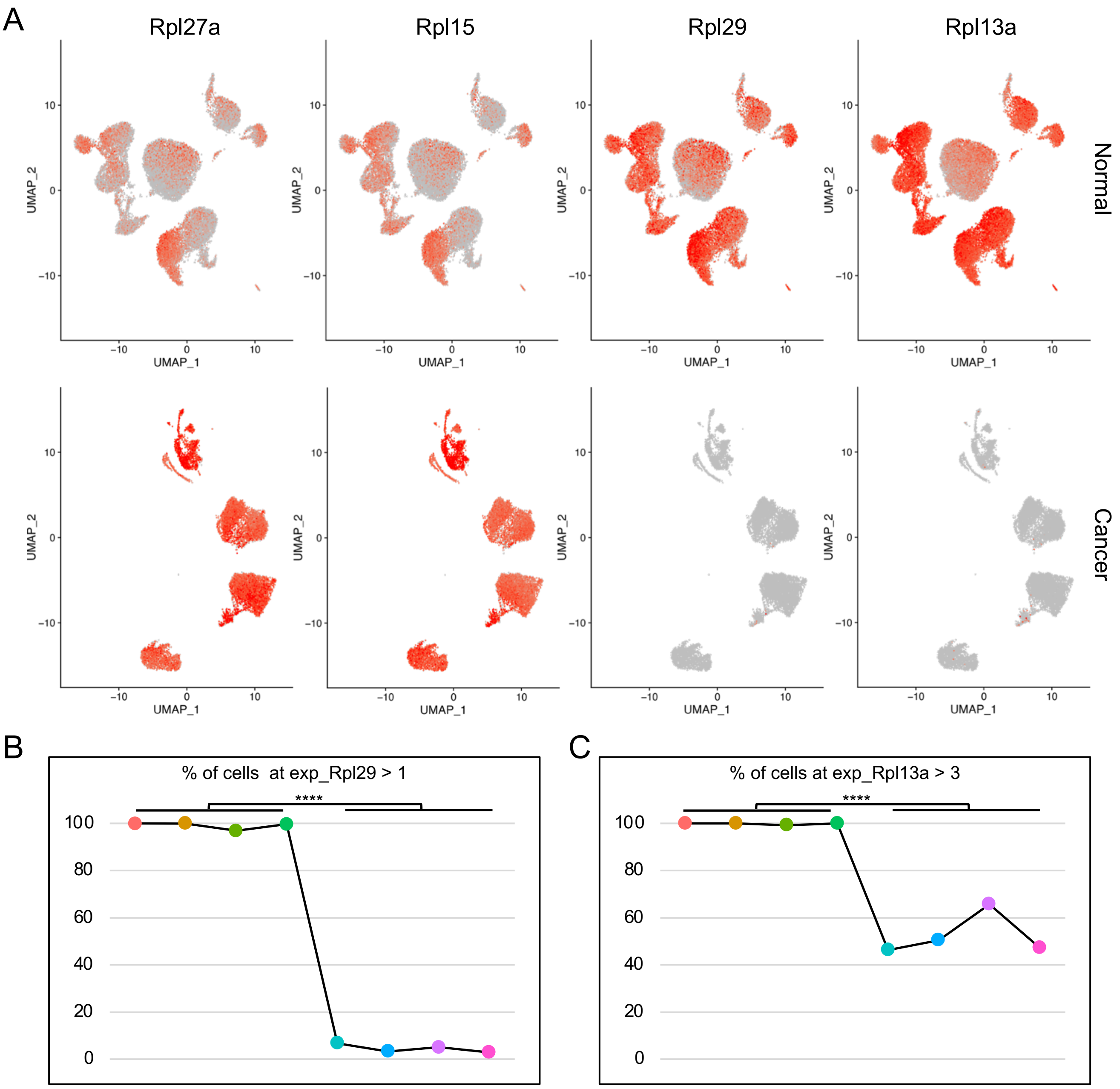

Supplementary Figure 5 | Expression levels of Rpl27a, Rpl15, Rpl29 and Rpl13a in normal mouse mammary epithelial cells and breast cancer cells. (**A**) Split UMAP visualization of Rpl27a, Rpl15, Rpl29 and Rpl13a in normal epithelial cells and cancer cells. Quantification of normal epithelial and cancer cells with Rpl29 expression at Rpl29 > 1 (**B**) and Rpl13a expression at Rpl13a > 3 (**C**). ****, $p < 0.0001$.

Figure S6

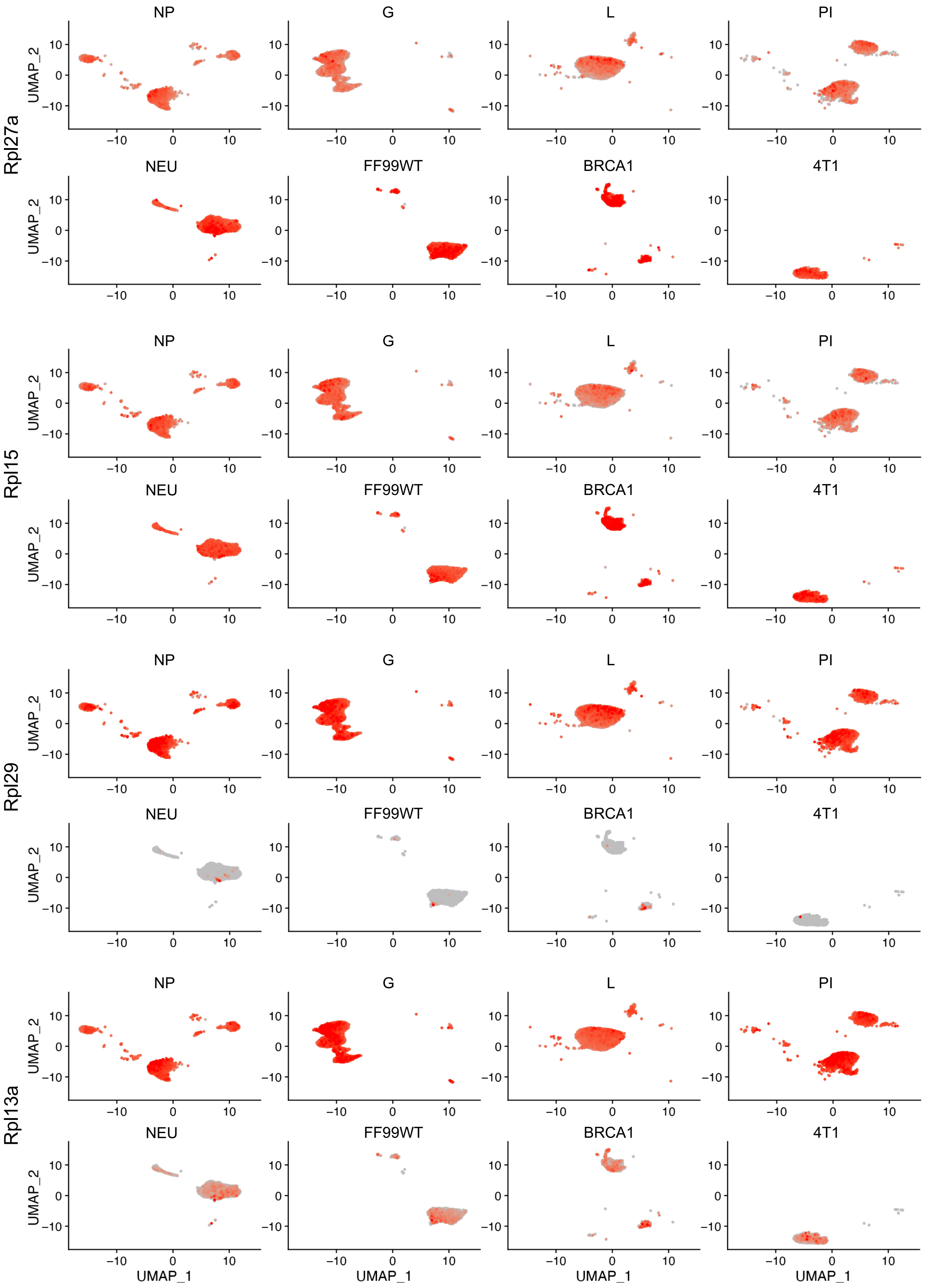

Supplementary Figure 6 | Expression levels of Rpl27a, Rpl15, Rpl29 and Rpl13a in normal mouse mammary epithelial cells of different developmental stages and breast cancer cells of different mouse models visualized by UMAPs.

Figure S7

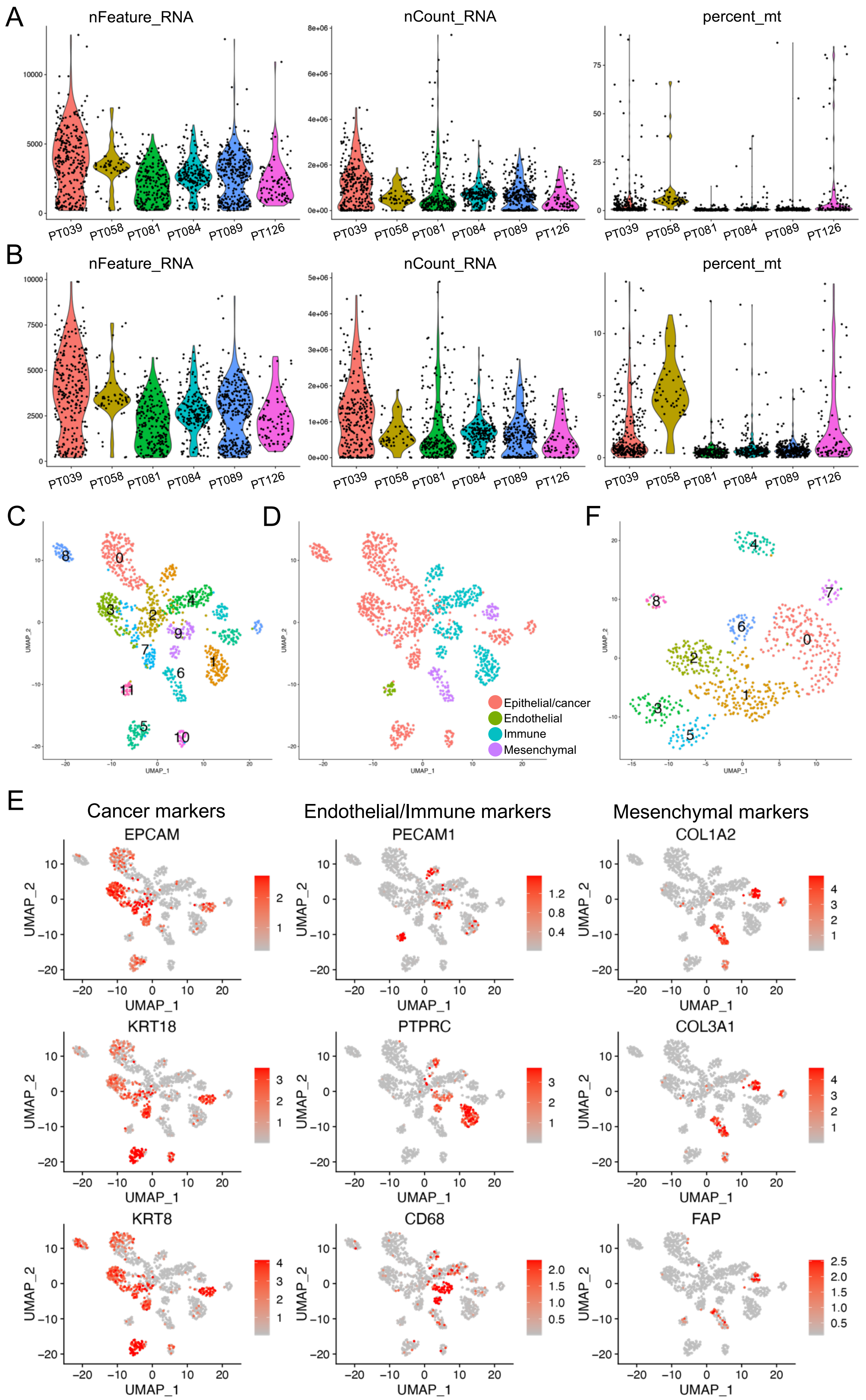

Supplementary Figure 7 | Quality control and cell type identification of scRNA-seq data on primary TNBC tumors. Cell qualities of each primary TNBC tumor sample before (**A**) and after QC (**B**) visualized by violin plots of the total number of genes detected each cell (nFeature_RNA), number of transcripts each cell (nCount_RNA), and percentage of mitochondrial genes of each cell (percent.MT). The integrated data of the primary TNBC tumors were clustered (**C**) and major cell types were identified (**D**, **E**). (**F**) Cancer cells were extracted and re-clustered.

Figure S8

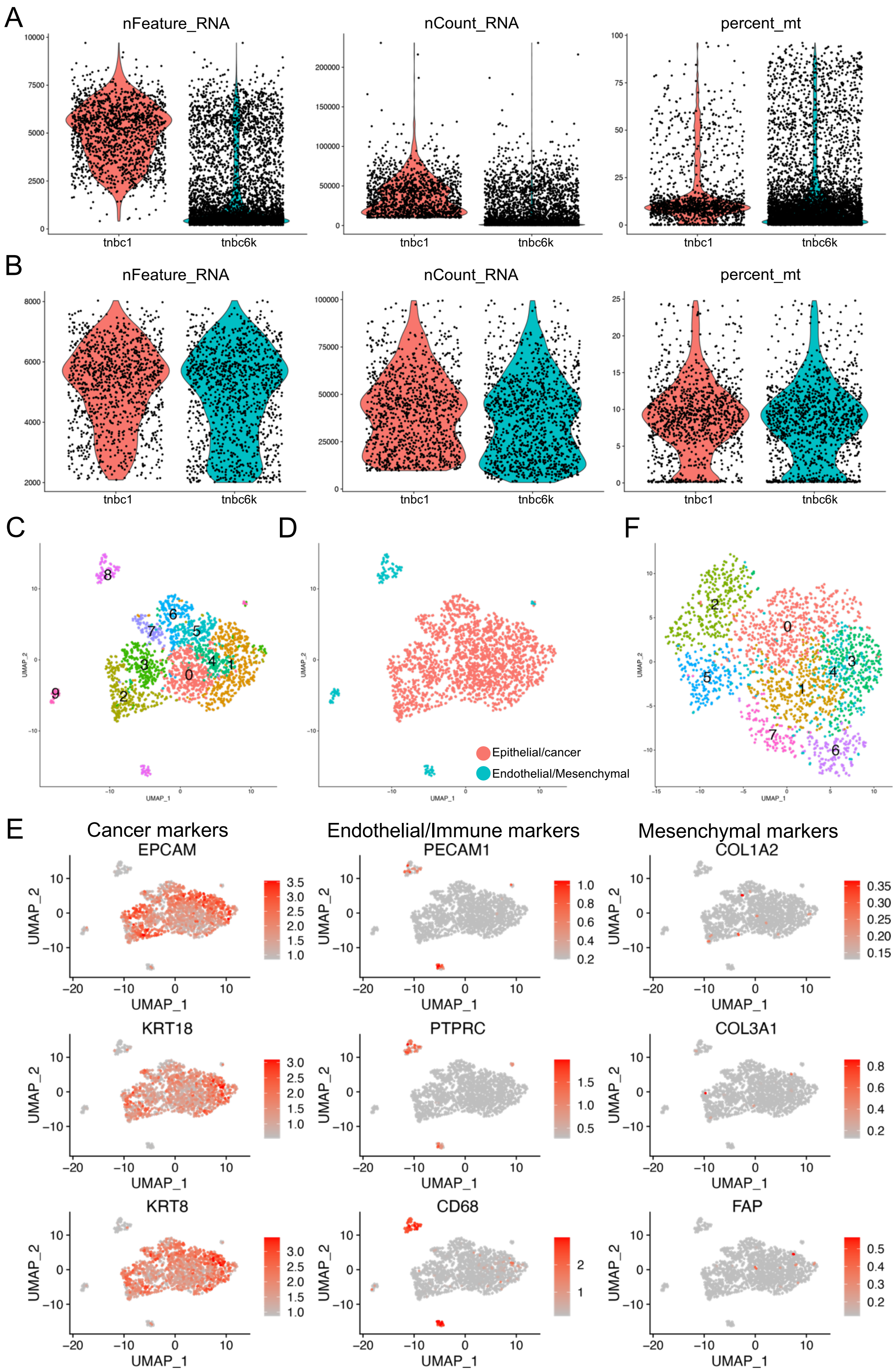

Supplementary Figure 8 | Quality control and cell type identification of scRNA-seq data on brain metastatic TNBC tumors. Cell qualities of two batches of scRNA-seq data on metastatic TNBC tumor before (**A**) and after QC (**B**) visualized by violin plots of the total number of genes detected each cell (nFeature_RNA), number of transcripts each cell (nCount_RNA), and percentage of mitochondrial genes of each cell (percent.MT). (**C**) Two batches of scRNA-seq data were integrated clustered. Cell types were identified (**D**) based on canonical cell type marker gene expression (**E**). (**F**) Cancer cells were subset and re-clustered.

Figure S9

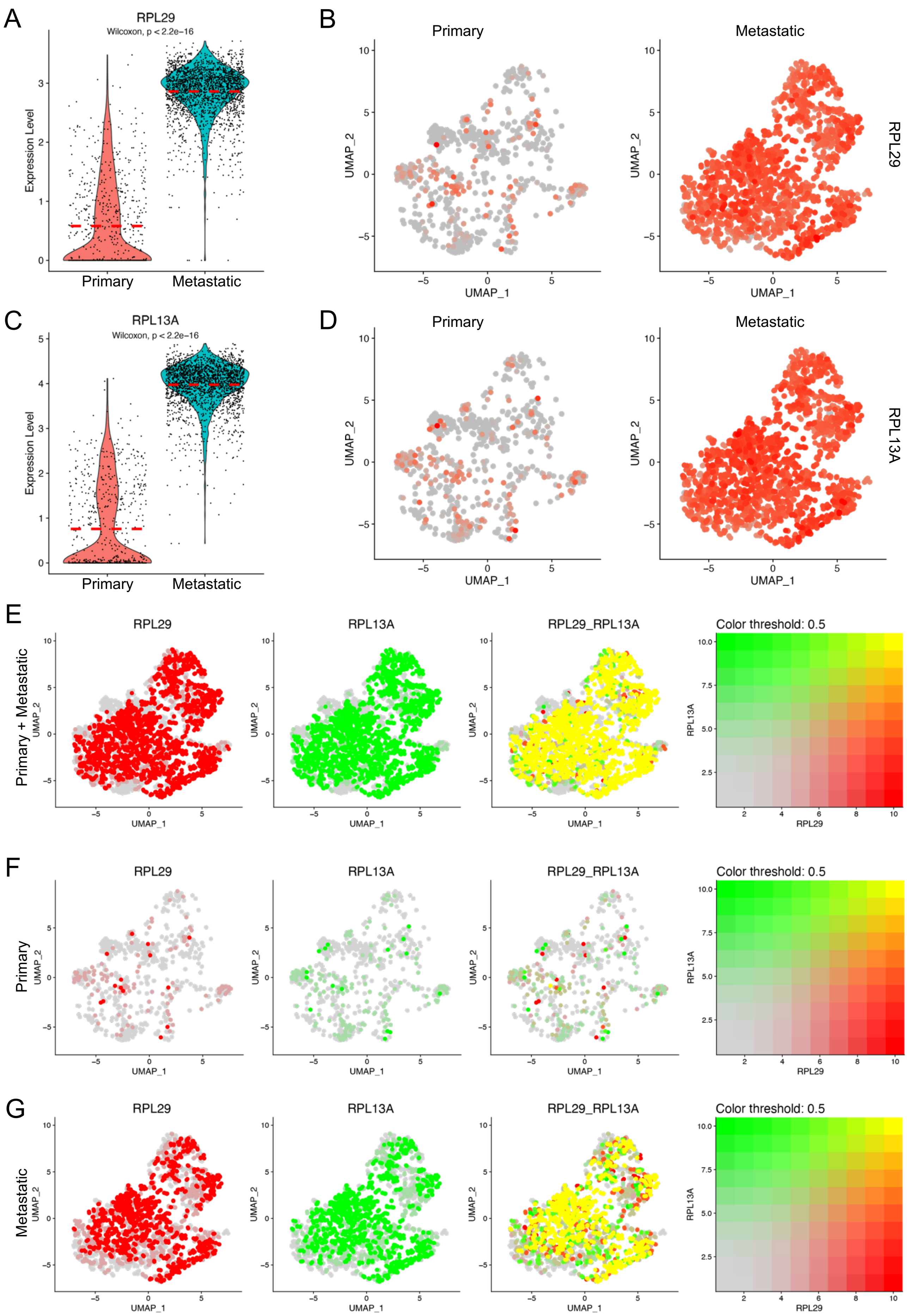

Supplementary Figure 9 | The expression of RPL29 and RPL13A in primary and metastatic TNBC cancer cells. Comparison of RPL29 and RPL13A expression in primary and metastatic TNBC cancer cells visualized by violin plots (**A**, **C**) and UMAPs (**B**, **D**). Transcription colocalization of RPL29 and RPL13A in the integrated TNBC cancer cells (**E**), primary TNBC cancer cells (**F**) and metastatic TNBC cancer cells (**G**).

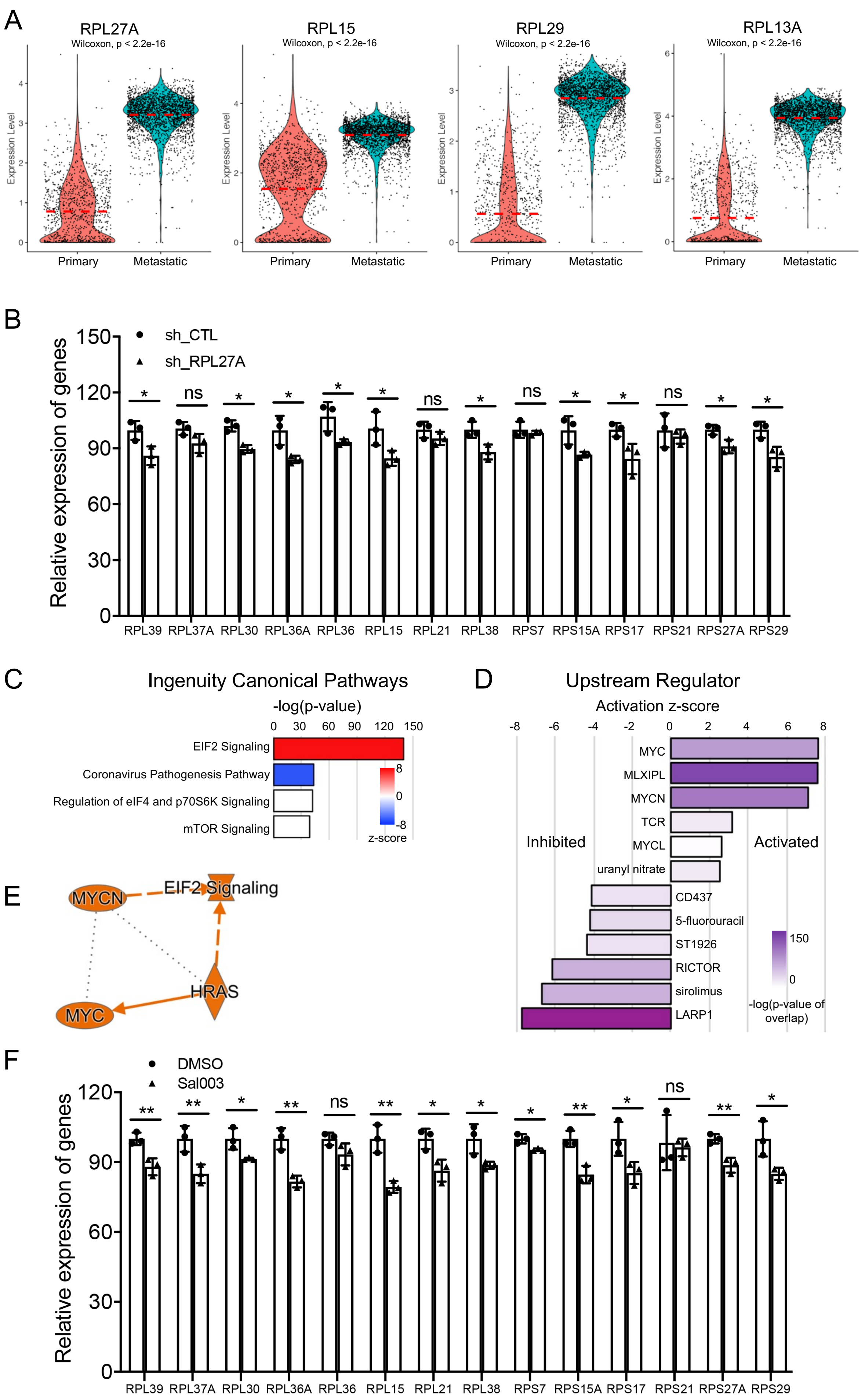

Supplementary Figure 10 | IPA analysis on the ribosome protein genes in metastatic cancer cells. (**A**) Relative expression of the RPL27A, RPL15, RPL29 and RPL13A in total cells of primary and metastatic TNBC tissues. (**B**) Relative expression of representative ribosome protein genes after RPL27A knockdown (**B**). (**C**, **D**) Bar plots showed the top Ingenuity Canonical Pathways (**C**) and Upstream Regulators (**D**) based on the upregulated ribosome protein genes in metastatic cancer cells. (**E**) Graphic regulatory networks of the Ingenuity Canonical Pathways (**C**) and Upstream Regulators (**D**). Color codes: Orange, upregulated. Color shapes: different molecular/gene types. Lines: solid lines, direct regulation; dash lines, indirect regulation. (**F**) Relative expression of representative ribosome protein genes after Sal003 treatment (**F**). *, $p < 0.05$; **, $p < 0.01$.
